# A Systematic Review of Ovarian Tissue Transplantation Outcomes by Ovarian Tissue Processing Size for Cryopreservation

**DOI:** 10.3389/fendo.2022.918899

**Published:** 2022-06-10

**Authors:** Ashley A. Diaz, Hana Kubo, Nicole Handa, Maria Hanna, Monica M. Laronda

**Affiliations:** ^1^ Stanley Manne Children’s Research Institute, Ann & Robert H. Lurie Children’s Hospital of Chicago, Chicago, IL, United States; ^2^ Feinberg School of Medicine, Northwestern University, Chicago, IL, United States

**Keywords:** fertility preservation, ovarian tissue cryopreservation, ovarian tissue transplantation, ovarian tissue size, oncofertility

## Abstract

Ovarian tissue cryopreservation (OTC) is the only pre-treatment option currently available to preserve fertility for prepubescent girls and patients who cannot undergo ovarian stimulation. Currently, there is no standardized method of processing ovarian tissue for cryopreservation, despite evidence that fragmentation of ovaries may trigger primordial follicle activation. Because fragmentation may influence ovarian transplant function, the purpose of this systematic review was (1) to identify the processing sizes and dimensions of ovarian tissue within sites around the world, and (2) to examine the reported outcomes of ovarian tissue transplantation including, reported duration of hormone restoration, pregnancy, and live birth. A total of 2,252 abstracts were screened against the inclusion criteria. In this systematic review, 103 studies were included for analysis of tissue processing size and 21 studies were included for analysis of ovarian transplantation outcomes. Only studies where ovarian tissue was cryopreserved (via slow freezing or vitrification) and transplanted orthotopically were included in the review. The size of cryopreserved ovarian tissue was categorized based on dimensions into strips, squares, and fragments. Of the 103 studies, 58 fertility preservation sites were identified that processed ovarian tissue into strips (62%), squares (25.8%), or fragments (31%). Ovarian tissue transplantation was performed in 92 participants that had ovarian tissue cryopreserved into strips (n = 51), squares (n = 37), and fragments (n = 4). All participants had ovarian tissue cryopreserved by slow freezing. The pregnancy rate was 81.3%, 45.5%, 66.7% in the strips, squares, fragment groups, respectively. The live birth rate was 56.3%, 18.2%, 66.7% in the strips, squares, fragment groups, respectively. The mean time from ovarian tissue transplantation to ovarian hormone restoration was 3.88 months, 3.56 months, and 3 months in the strips, squares, and fragments groups, respectively. There was no significant difference between the time of ovarian function’ restoration and the size of ovarian tissue. Transplantation of ovarian tissue, regardless of its processing dimensions, restores ovarian hormone activity in the participants that were reported in the literature. More detailed information about the tissue processing size and outcomes post-transplant are required to identify a preferred or more successful processing method.

**Systematic Review Registration:**

[https://www.crd.york.ac.uk], identifier [CRD42020189120].

## Introduction

According to the American Cancer Society, 927,910 women and 10,500 children were diagnosed with cancer in 2021 (1). Advancements in cancer treatments, such as chemotherapy and radiation, have led to an increased chance of survival in these patients. Specifically, the 5-year survival rate of women aged 15-39 and children under the age of 14 is 86.7% and 84%, respectively (2). Cancer survivors are interested in methods that would improve their quality of life after treatment (3).

Cancer treatments such as the alkylating agent cyclophosphamide are gonadotoxic and cause irreversible damage to the germ cells by triggering double-stranded DNA breaks leading to apoptosis (4). This extreme decline in germ cells directly impairs ovarian endocrine function, which has systemic effects in the body, such as the increased risk of osteoporosis, high blood pressure, cardiovascular disease, and decline in cognitive function (5). The American Society of Clinical Oncology has recommended that oncologists describe and offer fertility preservation to their patients (6). Fertility preservation methods include ovarian transposition, gonadal shielding during pelvic radiotherapy, egg cryopreservation, embryo cryopreservation, and ovarian tissue cryopreservation (OTC) (7). The latter of these methods is the only option currently available to preserve fertility for prepubescent since they do not produce mature gametes (7, 8). OTC preserves primordial follicles within ovarian cortical microenvironment (7). Four key components are involved in OTC: ovarian surgical procurement, ovarian tissue processing, tissue cryopreservation, and storage. A unique feature of OTC is that in the future, the patient can choose to reimplant the ovarian cortical tissue orthotopically or heterotopically for fertility purposes or to restore ovarian endocrine function (7). Not only has this fertility preservation method been used to benefit cancer patients, but also women who want to postpone fertility and menopause (7). In 1999, the first successful autotransplantation of frozen-thawed ovarian cortical tissue was performed, but it was not until 2006 when Meirow et al. reported the first live birth obtained from OTC (9, 10).

Reported in 2017, ovarian tissue transplantation (OTT) has resulted in over 130 births (11). A recent meta-analysis of three centers has stated a pregnancy rate of about 50% (12). Although a vast majority of participants (95%) have the return of endocrine function post-transplantation, the average duration of endocrine function of ovarian tissue after transplantation is approximately 2-5 years (13). It has been shown that the duration of the ovarian tissue function is correlated to its ovarian reserve (14). Additionally, in xenograft experiments there is a decline in primordial follicles *via* activation and apoptosis in human, bovine, and marmoset 3-days post-transplantation of ovarian tissue (14). A review from Roness et al. has outlined that every step of OTC/OTT from the participant’s initial reserve to transplantation site can impact the premature loss of the primordial follicles and impact ovarian tissue function (15). Studies done in mice and humans have shown that fragmentation of ovarian tissue stimulates follicle activation pathways, such as Hippo and PI3K-AKT (16, 17). Fragmenting the ovarian cortex during the tissue processing for OTC may activate primordial follicles and reduce the ovarian reserve. OTC has recently been designated as a nonexperimental procedure by the American Society for Reproductive Medicine (ASRM) (18). However, there is currently no standard method of processing ovarian tissue, which emphasizes the importance of examining if fragmentation impacts the function of transplanted ovarian tissue. In this systematic review, we sought (1) to identify the sizes and processing techniques used to cryopreserve ovarian tissue around the world, and (2) to examine the reported results of different sized ovarian cortical tissue in participants who have undergone OTT on functional longevity, hormone restoration, pregnancy, and live birth. This report examines different dimensions of cryopreserved ovarian cortical tissue on fertility and ovarian function post-transplantation.

## Materials and Methods

### Search Strategy

This systematic review followed the Preferred Reporting Items for Systematic Review and Meta-Analysis Protocols (PRISMA-P) guidelines and statement. This review’s protocol is registered on PROSPERO and is available on Centre for Reviews and Dissemination (CRD) website at https://www.crd.york.ac.uk (registration number #: CRD42020189120). All literature searches were conducted on the National Institute of Health (NIH) PubMed database (https://www.ncbi.nlm.nih.gov/pubmed/). We performed all searches until February 2022. The searches had no date restrictions, the document types included were case studies, multi-center studies, and articles that focused on the processing size of ovarian cortical tissue and reproductive outcomes in human patients who have undergone OTT. We included the following keywords in the search: “ovarian tissue cryopreservation”, “hormone restoration”, “live birth”, “success”, “output”, “size”, “fragments”, “strips”, “cubes”, “squares”, “slivers”, “processing”, and “transplantation”. A total of 14 searches were conducted. The parameters for the searches were: ((ovarian tissue cryopreservation), (ovarian tissue cryopreservation) AND ((hormone restoration) OR (pregnancy) OR (live birth) OR (success) OR (output)), (((ovarian tissue cryopreservation) AND((hormone restoration) OR (pregnancy) OR (live birth) OR (success) OR (output)) AND ((fragments) OR (strips) OR (squares) OR (cubes) OR (slices) OR (pieces) OR (slivers))), (((fragments) OR (strips) OR (squares) OR (cubes) OR (slices) OR (pieces) OR (slivers)) AND (Ovarian tissue processing)), ((((fragments) OR (strips) OR (squares) OR (cubes) OR (slices) OR (pieces) OR (slivers)) AND (Ovarian tissue processing)) AND (((Live birth) or (pregnancy) or (hormone restoration)))) AND (ovarian tissue cryopreservation), ((fragments) OR (strips) OR (squares) OR (cubes) OR (slices) OR (pieces) OR (slivers)) AND (Ovarian tissue cryopreservation)), (((fragments) OR (strips) OR (squares) OR (cubes) OR (slices) OR (pieces) OR (slivers)) AND (Ovarian tissue cryopreservation))) AND (((Pregnancy) or (live birth) or (success))), ((ovarian tissue cryopreservation) AND ((squares) OR (strips) OR (cubes) OR (slivers) OR (fragments) OR (pieces)), (((ovarian tissue cryopreservation) AND ((squares) OR (strips) OR (cubes) OR (slivers) OR (fragments) OR (pieces))) AND ((hormone function) OR (ovarian activity)), ((ovarian tissue cryopreservation) AND ((squares) OR (strips) OR (cubes) OR (slivers) OR (fragments) OR (pieces))) AND (restoration), (ovarian tissue cryopreservation) AND ((size) OR (fragments) OR (cubes) OR (strips) OR (slivers) OR (processing)), (ovarian tissue cryopreservation) AND (processing), (ovarian tissue cryopreservation) AND (size), (ovarian tissue cryopreservation) AND ((fragments) OR (strips) OR (cubes) OR (squares) OR (slivers)), respectively. The results were compiled, and duplicated results were removed. Titles and abstracts were manually reviewed to determine articles that met the inclusion/exclusion criteria. We presented a PRISMA flow diagram to layout the identification, screening, eligibility, and included studies for this review.

### Inclusion/Exclusion Criteria

In this systematic review, we had two subgroups: ovarian tissue processing for cryopreservation and transplanted frozen/thawed processed ovarian tissue. Studies focused on ovarian tissue processing for cryopreservation were gathered for the processing analysis, and studies that included outcomes of OTT were considered for the outcomes analysis. Only studies that contained participants who have undergone OTC regardless of age, diagnosis, and previous cancer treatment for fertility preservation and hormone restoration purposes were included for the outcomes analysis. Studies were excluded if ovarian tissue was used for other reasons than future fertility or hormone restoration purposes such as for experimental analysis. These included studies underwent further screening which focused on OTT outcomes. For the outcomes analysis, studies which contained participants who have undergone OTC and orthotopic auto transplantation regardless of age, diagnosis, and previous cancer treatment were included. Studies that contained participants who have only undergone OTC without a record of transplantation, non-human studies, ovarian tissue that was cultured/incubated in drugs before transplantation, heterotopic OTT, and OTT using donor ovaries were not included in this analysis.

### Study Selection

Duplicated articles were removed from the lists that were generated from the search strategies above. The titles and abstracts of the remaining articles were manually examined by three independent reviewers (AAD, NH, MTH) for inclusion and exclusion criteria based on the criteria of this study, previously mentioned. Reasons for exclusion and inclusion for all articles were recorded in the screening process. To avoid disagreements and bias in the study selection process, a fourth reviewer (HK) screened the title, abstracts, and full text of all articles for inclusion criteria. Articles that met the inclusion criteria were independently examined for data extraction by three reviewers (AAD, NH, MTH).

### Data Extraction

Three reviewers (AAD, NH, MTH) assessed the full-length articles that met the inclusion criteria to extract study measures. The reasons for exclusion were recorded for those articles that did not meet the inclusion criteria after full-article examination. Articles that met the inclusion criteria for OTC had the following data extracted: lab name, location of site, country of origin, number of participants, age of participant(s) at OTC, condition of participant(s), previous cancer treatment prior to OTC, date range of which tissue was processed, surgical technique of ovary removal, partial or whole ovary removal, methods used to process ovarian tissue during OTC, techniques used to process tissue, name of the ‘size’ of cortical ovarian tissue processed, dimensions of tissue pieces (length x width x thickness, mm), number of total tissue processed/cryopreserved, and cryopreservation technique (slow freeze, vitrification). Only articles that cryopreserved human ovarian tissue for future fertility or hormone purposes, explicitly stated methods of processing ovarian tissue, and had clear dimensions (length, width) of OTC tissue were assessed. Additional data were gathered for studies that met inclusion criteria for OTT outcomes analyses including: age of participant(s) at OTT, site of transplantation, number of total ovarian tissue pieces transplanted, beginning of ovarian hormone restoration (months), the longevity of ovarian function (months), the number of participants which underwent transplantation, number of participants that underwent assisted reproductive technology (ART) to conceive (yes/no, number of rounds, number of participants), number of participants that conceived spontaneously (yes/no, number of participants), the number of pregnancies, and the number of live births. Every participant in these studies were treated individually to avoid bias and to effectively assess the ovarian activity outcomes. The extracted data was verified by one reviewer (AAD) for validity and bias.

### Characterization of Size

The size of cryopreserved ovarian tissue was characterized into three categories based on dimensions of tissue pieces (length, width): strips, squares, fragments. Processed ovarian tissue were categorized as strips if the length (≥ 5 mm) and width had different measurements. Squares were determined to be ovarian tissue which had the same measurements for length (≥ 5 mm) and width (≥ 5 mm). Fragments were determined to be tissue which had the length and width < 5 mm. Only studies that included clear dimensions of the length and width of the processed ovarian tissue were included in this review.

### Outcome Measurements

For the outcomes analysis the following data were evaluated: dimensions of processed ovarian tissue (length, width, thickness), most common dimensions of ovarian tissue, area of tissue transplanted, pregnancy rate, live birth rate, time to resumption of ovarian function post-transplantation, and longevity of ovarian hormone function. Only articles that contain these data measurements were assessed. The most common dimension of ovarian tissue was defined by the greatest number of participants that have the length and width of the ovarian tissue pieces in common. The average area of tissue transplanted was determined by the average area of the size of ovarian tissue pieces and the average amount of total tissue transplanted. Fertility rate was calculated to be percentage of the number of participants that obtained pregnancy to the number of participants that attempted pregnancy. Live birth rate was calculated to be the percentage of the number of participants who obtained live birth to participants who attempted pregnancy. We defined ovarian function restoration as the time of decline of FSH levels to premenopausal state, an increase in estradiol levels, or to time of resumption of menses, if the study included all these factors the former was noted.

### Statistical Analysis

The number of tissues transplanted, and area of tissue transplanted were expressed as mean and range. The following parameters: age at OTC, age at OTT, and time of ovarian restoration from OTT were expressed as mean with standard deviations. To determine the differences between size of ovarian tissue and age at OTC, OTT, and ovarian restoration a One-way ANOVA was performed, P<0.05 were considered significantly different. Statistical analyses were performed using GraphPad PRISM.

### Risk of Bias

Two authors (AAD, HK) independently evaluated each article included in this systematic review for risk for bias. In this systematic review case reports, multi-center studies, and articles were assessed for quality assessment. For these platforms, the Joanna Briggs Institute Critical appraisal checklist was utilized to examine the clarity on the participant’s demographic characteristics, medical history, current clinical condition, intervention or treatment procedure, post-interventions, adverse/unanticipated events. Disagreements or conflicts on risk of bias assessment were resolved by a third reviewer (MML) **(** **Supplementary Tables 1, 2** **)**.

## Results

### Study Selection

**Figure 1:**
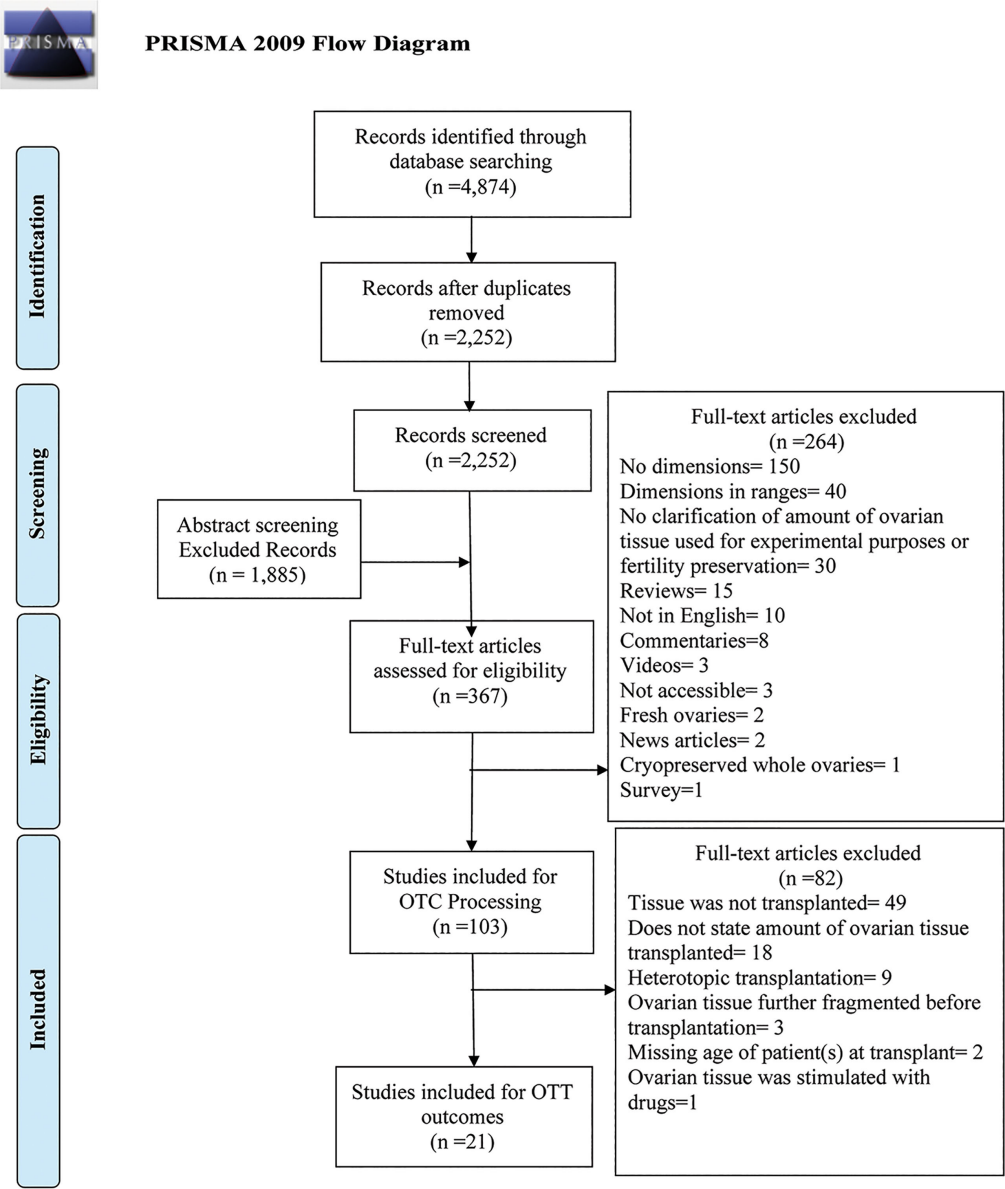
PRISMA Flow diagram for ovarian tissue cryopreservation and transplantation.

**Figure 1** represents the PRISMA Flow diagram that was constructed for this systematic review. This diagram shows the screening, identification, eligibility, and inclusion steps conducted for OTC processing and OTT outcomes analyses. Studies that focused on OTC were gathered for OTC processing research; these articles underwent further screening for OTT and for OTT outcomes analysis. A total of 4,874 results were identified in PubMed; after manual removal of duplicate results, a total of 2,252 results were assessed for screening against the OTC inclusion criteria (**Figure 1**).

Of the abstract screened, 367 results met the inclusion criteria for the tissue processing analysis. Two-hundred and sixty-four results were excluded with reasons **(** **Figure 1** **)**. A total of 103 studies specifying the methods of processing ovarian tissue for cryopreservation, dimensions of cryopreserved ovarian tissue pieces, and location of the site were identified and included in this review for the outcomes analysis **(** **Figure 1** **)**. As previously mentioned, the articles included for processing analysis were further screened against the inclusion criteria for OTT. Twenty-one articles that were used for processing analysis were included for OTT outcomes analysis in this review. A total of 82 articles were excluded from outcomes analysis with reasons **(** **Figure 1** **)**. In this systematic review, 103 studies that contained participants that underwent OTC were included **(** **Supplementary Table 3** **)**. Additionally, 21 studies described 92 participants who underwent OTC and autologous transplantation **(** **Supplementary Table 4** **)**.

### Ovarian Tissue Processing Sites

In this review, 58 unique sites published how ovarian tissue was processed for cryopreservation and tissue size categorized into three groups: strips, squares, and fragments (**Table 1**). There were 15, 3, and 10 unique length and width dimensions for strips, squares, and fragments, respectively. A total of 36 sites (62%) from 18 countries processed the ovarian tissue into strips (19–71) (**Table 1**). The most common dimension (length, width) for strips of 10 mm x 5 mm is processed in 19 different sites, with a range of 5-30 mm by 1-20 mm, (length, width). A total of 15 sites (25.8%) process ovarian tissue into squares, with the dimensions of 5 x 5 mm being the most common in 12 different sites and a range of 5-10 mm by 5-10 mm (length, width) (**Table 1**). Ovarian tissue was processed into squares in 15 sites from 11 countries (20, 65, 72–98). In total, 17 different sites (29.3%) from 12 countries process ovarian tissue into dimensions that were categorized into fragments (**Table 1**) (21, 62, 77, 99–122). Fragments with the dimensions of 2 x 2 mm (length, width) were the most common in 5 sites. The fragment dimensions ranged from 0.5-4 by 0.3-3 mm (length, width) (**Table 1**). Reported thicknesses ranged from 1 – 2 mm, and one site reporting a thickness of 5 mm (**Table 1**).

**Table 1:** Ovarian tissue cryopreservation processing sizes across different sites around the world.

Size	Dimensions length x width (mm^2^) ^thickness (*site*)^	Sites of OTC processing
**Strips**	161 x 5 ^a^ *^(a)^* 30 x 20 ^a^ *^(b)^* 20 x 10 ^b^ *^(c)^* 12 x 4 ^a^ *^(d)^* 10 x 5 ^a^ *^(d, e, f, g),^* ^b^ *^(d, h-p),^* ^d^ *^(q-u),^* ^e^ *^(v, w)^* 10 x 4 ^b^ *^(d)^* 10 x 3 ^a^ *^(d, x),^* ^b^ *^(u)^* 10 x 1^a^ *^(y)^* 8 x 5 ^a^ *^(l, z)^* 8 x 4 ^b^ *^(h, aa-dd)^* 6 x 4 ^d^ *^(ee)^* 5 x 4 ^b^ *^(ff)^* 5 x 3 ^b^ *^(gg),^* ^d^ *^(ee)^* 5 x 2 ^b^ *^(hh)^* 5 x 1 ^b^ *^(ii, jj),^* ^g^ *^(ff)^*	a. University Paul Sabatier, Toulouse, France. b. Sheba Medical Center, Sackler School of Medicine, Tel-Aviv University, Tel-Aviv, Israel. c. St. Luke's Hospital, St. Louis, Missouri, USA. d. Cliniques Universitaires Saint-Luc, Université Catholique de Louvain, Brussels, Belgium. e. Medical University of Vienna, Vienna, Austria. f. University Medical Centre Ljubljana, Ljubljana, Slovenia. g. Institute University Dexeus, Barcelona, Spain. h. Beijing Obstetrics and Gynecology Hospital, Capital Medical University, Beijing, China. i. Royan Institute for Reproductive Biomedicine, ACECR, Tehran, Iran. j. Israel and Sackler Faculty of Medicine, Tel Aviv University, Tel Aviv, Ramat Aviv, Israel k. Leiden University Medical Center, Leiden, the Netherlands. l. Radboud University Medical Center, Nijmegen, The Netherlands. m. Ankara University Faculty of Medicine, Ankara, Turkey. n. Christie Hospital, Manchester, UK. o. University of Pennsylvania, Philadelphia, USA. p. Children's National Hospital, Washington, D.C., USA q. Hôpital Jean-Verdier, Hôpitaux Universitaires Paris-Seine-Saint-Denis, Assistance Publique, Hôpitaux de Paris, Bondy, France. r. Lis Maternity Hospital, Tel Aviv Sourasky Medical Center, Tel Aviv, Israel. s. Chaim Sheba Medical Center, Tel Hashomer, Israel. t. Cleveland Clinic, Cleveland, Ohio, USA. u. University of Bologna, S Orsola-Malpighi Hospital of Bologna, Italy. v. New York Medical College, New York, New York, USA. w. Naval Medical Center San Diego, San Diego, California, USA x. University Medical Center Utrecht, Utrecht, The Netherlands. y. University of Valencia, Valencia, Spain. z. Centre for Reproductive Medicine, UZ Brussel, Brussels, Belgium. aa. Heinrich-Heine-University, Düsseldorf, Germany. bb. University Women's Hospital Düsseldorf, Düsseldorf, Germany. cc. Bern University Hospital, Bern, Switzerland. dd. Medical University of Bonn, Bonn, Germany. ee. Assistance Publique-Hôpitaux de Paris Saint Louis Hospital, Paris, France. ff. The Juliane Marie Centre for Women, University Hospital of Copenhagen, University ofCopenhagen, Rigshospitalet-Copenhagen, Denmark. gg. Marianna University School of Medicine, Kawasaki City, Japan. hh. Shahid Sadoughi University of Medical Sciences, Yazd, Iran. ii. Royal Women's Hospital, Parkville, Victoria, Australia. jj. Hadassah Hebrew University Hospital, Jerusalem, Israel. kk. Rose Ladies Clinic, Tokyo, Japan. ll. Monash IVF, Melbourne, Victoria, Australia. mm. Ghent University Hospital, Ghent, Belgium. nn. University of Torino, S. Anna Hospital, Torino, Italy. oo. Seoul National University Bundang Hospital, Seongnam, Korea. pp. Oslo University Hospital, Oslo, Norway. qq. AVA-PETER Fertility Clinic, Saint-Petersburg, Russia. rr. University of Kansas Medical Center, Kansas City, Kansas, USA. ss. Erasme Hospital, Université Libre de Bruxelles, Brussels, Belgium. tt. Aarhus University, Aarhus, Denmark. uu. Eulji University School of Medicine, Seoul, South Korea vv. Gameta Hospital, Lodz, Poland. ww. Monash Medical Centre, Clayton, Victoria, Australia. xx. University of Cologne, Cologne, Germany yy. Medical University of Innsbruck, Innsbruck, Austria. zz. Erlangen University Hospital, Friedrich-Alexander University of Erlangen-Nuremberg, Erlangen,Germany. aaa. Karolinska University Hospital, Stockholm, Sweden. bbb. Hospital Center São João, Porto, Portugal. ccc. McGill University Health Center, McGill University, Montreal, Quebec, Canada ddd. University of Oxford, Oxford, UK. eee. Johannes Gutenberg University, Mainz, Germany. fff. Département de Biologie de la Reproduction, CHU Montpellier, Univ Montpellier, France.
**Squares**	12 x 12 ^b^ *^(kk)^* 10 x 10 ^b^ *^(gg),^* ^c^ *^(c),^* ^d^ *^(gg, kk)^* 5 x 5 ^a^ *^(ff),^* ^b^ *^(ii, ff, mm-rr),^* ^d^ *^(ff, ss-vv),^* ^e^ *^(ss)^*	
**Fragments**	4 x 3 ^a^ *^(ff)^* 4 x 2 ^b^ *^(ii, ww),^* ^e^ *^(xx)^* 3 x 3 ^a^ *^(ff),^* ^b^ *^(yy, zz)^* 3 x 2 ^d^ *^(ss)^* 3 x 1 ^f^ *^(aaa)^* 2 x 2 ^a^ ^*(ff, w, bbb),* b (*ccc*), e (*d*)^ 2 x 1 ^a^ ^(zz), b (*dd*), f (*ddd*)^ 1 x 1 ^a (*zz, eee*)^ 0.5 x 0.5 ^b^ *^(fff)^* 0.5 x 0.3 ^a^ *^(r)^*	

a thickness no mentioned.

b thickness of 1 mm.

c thickness of 1-1.5 mm.

d thickness of 1-2 mm.

e thickness of 2 mm.

f thickness of 5 mm.

Of the 58 unique OTC processing sites, 8 (13.8%) cut ovarian tissue into different dimensions within the same size categories, 6,1, and 2 sites for strips, squares, and fragments, respectively (**Table 1**). Additionally, 9 sites (15.5%) processed ovarian tissue into different sizes (**Table 2**).

**Table 2:** Processing sites that cut ovarian tissue into different sizes.

Size of processed ovarian tissue	Site(s)	Dimensions length x width (mm^2^)
**All three sizes**	The Juliane Marie Centre for Women, University Hospital of Copenhagen, University of Copenhagen, Copenhagen, Denmark.	Strips: 5 x 4, 5 x 1 Squares: 5 x 5 Fragments: 4 x 3, 3 x 3, 3 x 2, 2 x 2
	Royal Women’s Hospital, Parkville, Victoria, Australia.	Strips: 5 x 1 Squares: 5 x 5 Fragments: 2 x 2, 3 x 3, 4 x 3
**Strips and fragments**	Cliniques Universitaires Saint-Luc, Université Catholique de Louvain, Brussels, Belgium.	Strips: 12 x 4, 10 x 5, 10 x 4, 10 x 3 Fragments: 2 x 2
	Lis Maternity Hospital, Tel Aviv Sourasky Medical Center, Tel Aviv, Israel.	Strips: 10 x 5 Fragments: 0.5 x 0.3
	University Medical Center Utrecht, Utrecht, The Netherlands.	Strips: 10 x 3 Fragments: 2 x 2
	Medical University of Bonn, Bonn, Germany.	Strips: 8 x 4 Fragments: 2 x 1
**Strips and squares**	St. Luke’s Hospital, St. Louis, Missouri, USA.	Strips: 20 x 10 Squares: 10 x 10
	Marianna University School of Medicine, Kawasaki City, Japan.	Strips: 5 x 3 Squares: 10 x 10
**Squares and fragments**	Erasme Hospital, Université Libre de Bruxelles, Brussels, Belgium.	Squares: 5 x 5 Fragments: 3 x 2

To determine if there is a correlation between age at OTC, participant diagnosis or ovarian tissue procurement method and the chosen size for tissue processing, additional data was extracted from articles where sites reported using multiple tissue processing sizes. Two sites (3.4%) cryopreserved ovarian tissue into all three sizes (**Table 2**.) The Juliane Marie Centre for Women at University Hospital of Copenhagen in Copenhagen, Denmark reported cutting tissue into the following dimensions (1): strips: 5 x 4, 5 x 1 (2), squares: 5 x 5 (3), fragments: 4 x 3, 3 x 3, 3 x 2, 2 x 2 mm^2^ (length, width) (**Table 2**). This site processed a 9-year-old Ewing’s sarcoma participant’s ovarian tissue into 5 x 4 mm^2^ (64). Six galactosemia participants at this site had one ovary removed for fertility preservation at the ages of 1.7, 0.9, 4.5, 0.3, 2.9, and 11.7 and ovarian tissue was cut into the dimensions: 5 x 5, 4 x 3, 3 x 3, 3 x 2, 2 x 2, and 2 x 2 mm^2^, respectively (77). Additionally at this site, 25 leukemia participants had 5 x 1 mm^2^ strips cryopreserved (71). The Royal Women’s Hospital in Victoria, Australia also reported processing ovarian tissue into multiple different sizes (1): strips: 5 x 1 (2), squares: 5 x 5 (3), fragments: 2 x 2, 3 x 3, 4 x 3 mm^2^ (**Table 2**). Over 40 participants at this site had ovarian tissue processed into squares at the mean age of 26 (range 17.8 – 40.7), these participants had wide range of diagnoses (79). Also at this site 17 participants that had a mean age of 27.4 (range 17.8 – 35.9) at OTC had their ovarian tissue processed and cryopreserved into 5 x 1 mm^2^ strips (68). The Royal Women’s Hospital processed 9 participants (mean age at OTC= 20.6, range 18-31) ovarian tissue into 4 x 2 mm^2^ fragments, these participants had various from of diseases (120).

In total 4 sites (6.9%) cut ovarian tissue into strips and fragments. The Cliniques Universitaires Saint-Luc in Brussels, Belgium, reported processing ovarian tissue into strips and fragments with the following dimensions: 12 x 4, 10 x 5, 10 x 4, 10 x 3, and 2 x 2 mm^2^ (**Table 2**). At this site, 6 participants with different diagnoses and a mean age of 23.8 (range 21-28) underwent partial or whole oophorectomy had 12 x 4 mm^2^ strips cryopreserved (22). A 23-year-old sickle cell anemia participant, at the Cliniques Universitaires Saint-Luc, that underwent a unilateral oophorectomy for fertility preservation had 10 x 5 mm^2^ ovarian tissue strips cryopreserved (29). Four participants, with the mean age of 23.5 (range 21-28) at OTC, had their ovarian tissue was cut into 10 x 4 mm^2^ strips for cryopreservation (123). A 22-year-old participant at this site had 10 x 3 mm^2^ ovarian strips cryopreserved. Additionally at this site, 2 participants (17 and 25 years old at OTC) had fragments cryopreserved in the dimensions of 2 x 2 mm^2^ (22). In the Lis Maternity Hospital at Tel Aviv Sourasky Medical Center in Tel Aviv, Israel, 93 participants (average at OTC 15.4, range 0–25) with a wide range of diagnosis at OTC, had 0.5 x 0.3 mm^2^ fragments cryopreserved (**Table 2**) **(** 116). Also at this site, an 18 year old participant with Hodgkin’s disease had ovarian tissue cut into 10 x 5 mm^2^ strips for OTC (43). At University Medical Center Utrecht in Utrecht, The Netherlands, 10 participants with different diagnoses underwent unilateral oophorectomy for fertility preservation and had 10 x 3 strips and 2 x 2 mm^2^ fragments cryopreserved (52) (**Table 2**).

Two additional sites report processing tissue into strips and squares (3.4%). St. Luke’s Hospital in St. Louis, Missouri, USA cryopreserves participants’ ovarian tissue into 20 x 10 mm^2^ strips and 10 x 10 mm^2^ squares (20) (**Table 2**). From 1997 to 2007, slow freezing was this site’s main method of cryopreservation and therefore cut ovarian tissue into 20 x 10 mm^2^ strips (20). This site changed its method of cryopreservation to vitrification in 2007. From 2007 to 2017, participants at this site had 10 x 10 mm^2^ squares cryopreserved for fertility preservation (20). In Japan, the Marianna University School of Medicine uses 5 x 3 mm^2^ strips and 10 x 10 mm^2^ squares for participants with various diagnosis at OTC (17, 73) (**Table 2**).

Of the 58 processing sites 1 site uses both squares and fragments as its size for cryopreserving ovarian tissue. Erasme Hospital in Brussels, Belgium cryopreserves tissue as 5 x 5 mm^2^ squares, which has been used for over 200 participants (age range 0-27) with different diagnoses at OTC, and regardless of partial or whole ovary removal (89) (**Table 2**). Additionally, a 13-year-old sickle cell anemia participant at this site had 3 x 2 mm^2^ fragments ovarian tissue cryopreserved for fertility preservation (100).

### Characteristics of OTT Participants

**Figure 2:**
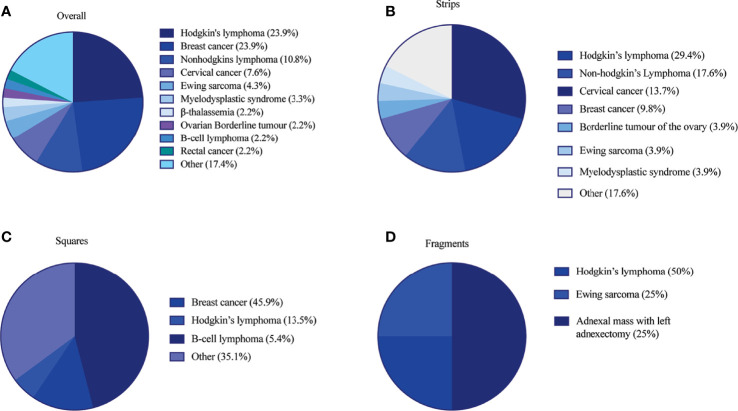
Participant diagnosis at time of OTC in **(A)** overall patient population **(B)** strips **(C)** squares and **(D)** fragments group. Diagnoses in the other category included the following in (1) overall: acute lymphocytic leukemia, adnexal mass with left adnexectomy, aplastic anemia, autoimmune vasculitis, choriocarcinoma, colorectal cancer, endometrial cancer, granulomatosis with polyangiitis, leukemia, neuroendocrine tumor, ovarian cancer, Schwachman-diamond syndrome, sickle cell anemia, synovial sarcoma of the lung and pelvic sarcoma, systemic lupus erythematosus, T-cell lymphoma (2), strips: acute lymphocytic leukemia, aplastic anemia, β-thalassemia, colorectal cancer, endometrial cancer, rectal cancer, Schwachman-diamond syndrome, sickle cell anemia, systemic lupus erythematosus, and (3) squares: autoimmune vasculitis, β-thalassemia, choriocarcinoma, Ewing’s sarcoma, granulomatosis with polyangiitis, leukemia, myelodysplastic syndrome, neuroendocrine tumor, non-Hodgkin’s lymphoma, ovarian cancer, rectal cancer, synovial sarcoma of the lung and pelvic sarcoma, T-cell lymphoma (21, 22, 25, 34, 37, 44, 57, 59, 61, 64, 66, 78, 84, 91–93, 95, 111, 115, 118, 124).

**Figure 3:**
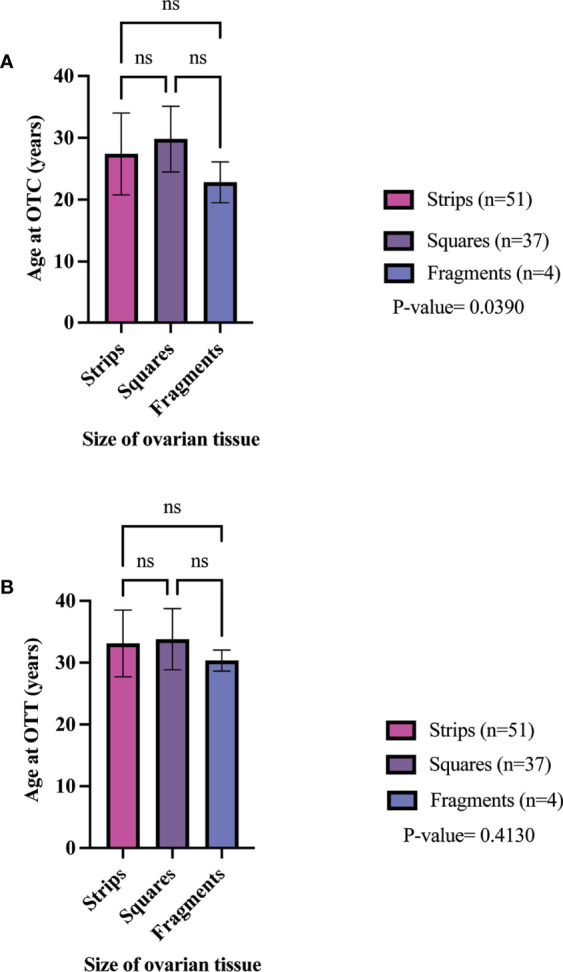
Age of participant at OTC **(A)** and OTT **(B)** in different size cryopreserved and transplanted ovarian tissue. P-values greater than 0.05 were considered not significantly different (ns) (21, 22, 25, 34, 37, 44, 57, 59, 61, 64, 66, 78, 84, 91–93, 95, 111, 115, 118, 124).

**Figure 4:**
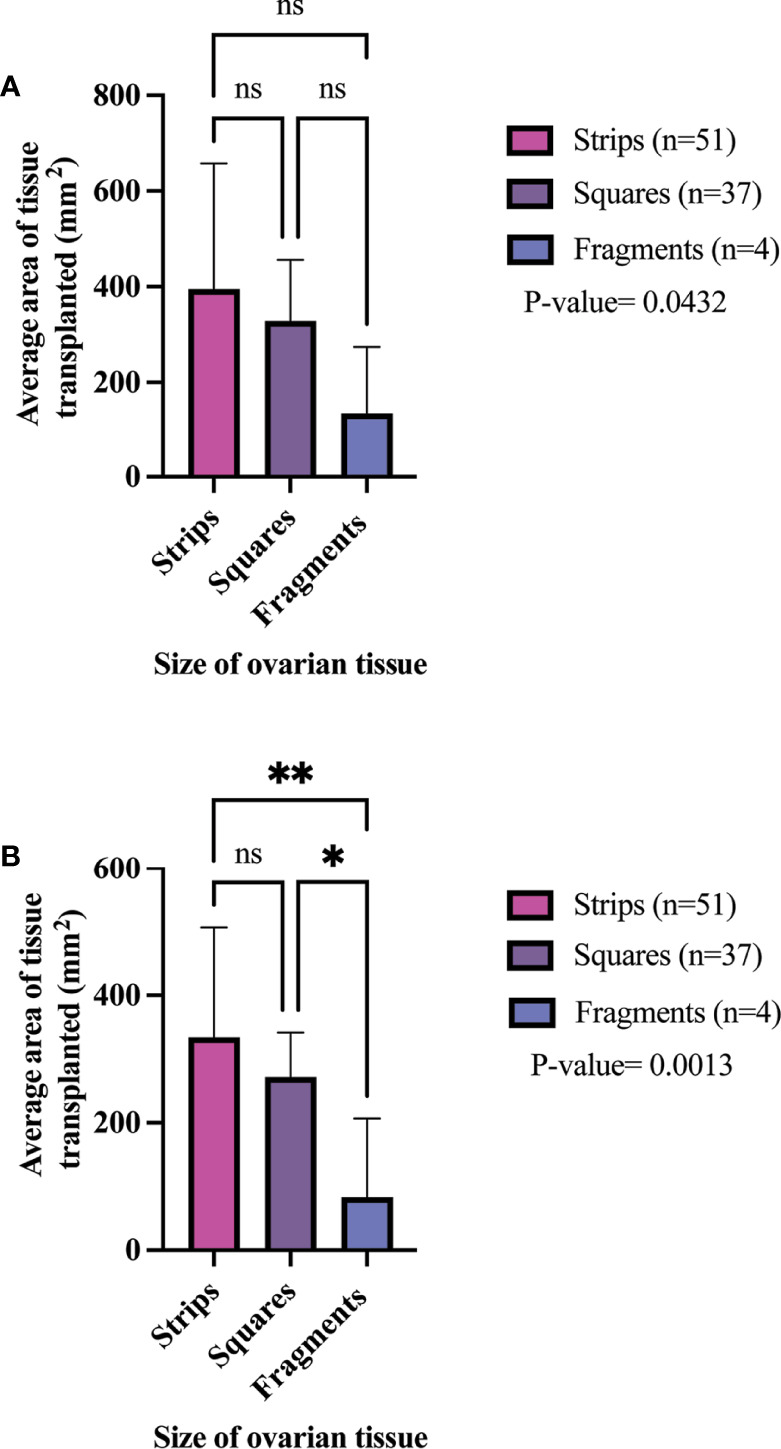
Total average area of tissue transplanted **(A)** and per 1^st^ OTT **(B)** in different size cryopreserved and transplanted ovarian tissue. P-values greater than 0.05 were considered not significantly different (ns) P-values greater than 0.05 were considered not significantly different (ns).P-values less than 0.05 (*) and 0.005(**) were considered significantly different. (21, 22, 25, 34, 37, 44, 57, 59, 61, 64, 66, 78, 84, 91–93, 95, 111, 115, 118, 124).

The clinical metadata for participants that were included in this systematic review is detailed in **Supplementary Table 4**.** **A total of 92 unique participants who underwent OTT after OTC with the goal of having a biological child or restoring hormones were included in this analysis (21, 22, 25, 34, 37, 44, 57, 59, 61, 64, 66, 78, 84, 91–93, 95, 111, 115, 118, 124). Overall, 51, 37, and 4 participants had their ovarian tissue processed into strips, squares, and fragments, respectively (**Table 3**). The three most predominant conditions that were present in the total participant population were Hodgkin’s lymphoma (23.9%), breast cancer (29.3%), and other conditions (17.4%) (**Figure 2A**). In the strips and fragment participant populations, Hodgkin’s lymphoma was the most prevalent diagnosis **(** **Figures 2B, D**). Additionally, the most prevalent condition for participants whose ovaries were processed into squares was breast cancer (**Figure 2C**). The mean age at OTC was 27.4, 29.8, and 22.8 years for participants whose ovaries were processed into strips, squares, and fragments, respectively (**Figure 3A**). The mean age at the time of OTT was 33.1, 33.8, and 30.3 for strips, squares, and fragments groups, respectively (**Figure 3B**). There was statistically significant difference in the age at the time of OTC (P-value= 0.0390) between all three sizes, but no significant difference between two sizes. Furthermore, there was no statistically significant difference between the age at the time OTT (P-value= 0.4130) in the three different size ovarian tissue pieces, respectively (**Figures 3A, B**). Although all groups had at least one participant with previous cancer treatment before OTC, the strips group had the greatest number of participants in this subgroup (n=21) (**Table 3**). The ovarian tissue was most often processed into the dimensions of 6 x 4 mm^2^, 5 x 5 mm^2^, and 2 x 2 mm^2^ (length x width) in the strips, squares, and fragments groups, respectively prior to OTT (**Table 3**). Eighteen participants underwent a second OTT 7, 10, and 1 strips, squares, and fragments groups, respectively and one participant underwent a third OTT (**Table 3**). A total of 51 participants whose ovarian tissue was processed into strips, 9 into squares, and 3 into fragment were included for analysis because information on hormone restoration and longevity was reported. The average area of tissue per first OTT was 334.0 mm^2^, 271.8 mm^2^, and 123.8 mm^2^ for strips, squares, and fragments groups, respectively. Overall, the total average area of tissue transplanted was 394.58 mm^2^, 328.4 mm^2^, and 134.8 mm^2^ for strips, squares, and fragments groups, respectively (**Table 3**). There was a statistically significant difference between the total and first average area of tissue per OTT in all three size ovarian tissue pieces. (P-value= 0.0432 and 0.0013, respectively) Additionally, there was not a statistically significant difference in total and first average area of tissue per OTT between two groups (**Table 3**; **Figures 4A, B**).

**Table 3:** OTC/OTT Participant characteristics for fertility and hormone outcomes (21, 22, 25, 34, 37, 44, 57, 59, 61, 64, 66, 78, 84, 91–93, 95, 111, 115, 118, 124).

Size of processed ovarian tissue	Strips	Squares	Fragments
**Range Dimensions of processed ovarian tissue Length x Width (**mm^2^ **)**	6-10 x 2-5	5 x 5	2-3 x 1-2
**Most common dimension Length x Width (**mm^2^ **), (n=67)**	6 x 4 (n = 28)	5 x 5 (n = 37)	2 x 2 (n = 2)
**No. of OTC/OTT participants**	51	37	4
**Method of cryopreservation** **(Slow freeze, Vitrification)**	Slow freeze: 50 Vitrification: 0	Slow freeze: 37 Vitrification: 0	Slow freeze: 4 Vitrification: 0
**Mean Age at OTC** **Years ± SD, Range**	27.4 ± 6.6, 9-40	29.8 ± 5.31, 15.4-38	22.8 ± 3.3, 18-25
**Mean Age at OTT** **Years ± SD, Range**	33.1 ± 5.3, 13.6-41.9	33.8± 4.95, 27-43	30.3 ± 1.7, 28-32
**No. of participants with previous treatment prior to OTC**	21	2	1
**Average number of tissues transplanted, ± SD, Range** **(1^st^, 2^nd^, 3^rd^)**	1^st^: 11.5 ± 7.8, 2-46 2^nd^: 11.7 ± 4, 8-16 (n=7)	1^st^:10.9 ± 2.8, 6-17 2^nd^: 8.3 ± 4.3, 3-16 (n=10)	1^st^: 21.8 ± 30.3, 4-67 2^nd^: 20 (n=1) 3^rd^: 49 (n=1)
**Av. Area of tissue transplanted (mm^2^) ± SD, Range, mm^2^** **(Total, 1^st^, 2^nd^, 3^rd^)**	Total: 394.58 ± 262.7, 40 – 1152 1^st^: 334.0 ± 172.5, 40-920 2^nd^: 385.75 ± 100.7, 216-550 3^rd^: -	Total: 328.4 ± 128, 150 -750 1^st^; 271.8 ± 69.9, 150-425 2^nd^: 207.5 ± 108, 75-400 mm^2^ 3^rd^: -	Total: 134.8 ± 139.8, 12-268 1^st^; 83 ± 123.8, 12-268 2^nd^: 60 (n=1) 3^rd^:147 (n=1)

### Ovarian Tissue Processing Size and Pregnancy Outcomes

52.2% (48/92) of all participants who were reported to undergo OTT were also monitored for pregnancy attempts using assisted reproductive technologies (ART) or spontaneously (21, 22, 25, 34, 37, 44, 57, 59, 61, 64, 66, 78, 84, 91–93, 95, 111, 115, 118, 124). A total of 16, 11 and 3 participants with strip, square, and fragment ovarian tissue pieces attempted pregnancy (**Table 4**). The pregnancy rates were 81.3, 45.5, and 66.7% for strip, square, and fragment ovarian tissue pieces (21, 22, 25, 34, 37, 44, 57, 59, 61, 64, 66, 78, 84, 91–93, 95, 111, 115, 118, 124).

**Table 4:** Pregnancy and live birth outcome after transplantation per 1^st^ transplant (21, 22, 25, 34, 37, 44, 57, 59, 61, 64, 66, 78, 84, 91–93, 95, 111, 115, 118, 124).

Size of processed ovarian tissue (No. of OTT Participants)	Participants who attempted to become pregnant n, (%)	Pregnancy rate n, (%)	Number of Pregnancies	Live birth rate n, (%)	Number of Live Births
**Strips** (n = 51) Total ART spontaneous	16^g^/51, (31.4) 6 13	13^h^/16, (81.3) 4 11	14 4 10^i^	9^j^/16, (56.3) 2 8	12^k^ 2 10
**Squares** (n = 37) Total ART spontaneous	11/37, (29.7) 6 5	5/11, (45.5) 2 3	5 2 3	2/11, (18.2) 1 1	2 1 1
**Fragments** (n = 4) Total ART spontaneous	3/4, (75) 0 3	2/3, (66.7) 0 2	2 0 2	2/3, (66.7) 0 2	2 0 2

^g^Three participants in the strips category used both ART and spontaneous methods to attempt pregnancy.

^h^Two participants in the strips category that used both ART and spontaneous methods to attempt pregnancy obtained pregnancy with both methods, one participant in the strips category that used both ART and spontaneous methods to attempt pregnancy obtained pregnancy only with spontaneous methods.

^i^One participant in the strips category that used both ART and spontaneous methods to attempt pregnancy obtained pregnancy only with spontaneous methods, had an ongoing pregnancy.

^j^One participant in the strips category obtained a live birth from both ART and spontaneous methods. ^k^ Two participants in the strips category had twins.

In the strips group, 6 participants used ART methods, 13 participants used spontaneous methods, and 3 participants used both methods to attempt pregnancy (25, 34, 37, 44, 57, 59, 61, 63, 64). Overall, there were 14 pregnancies in this group; 4 pregnancies resulted from ART, and 10 were obtained spontaneously. Of the three strips participants that used both methods to attempt pregnancy, 2 participants achieved pregnancy with ART and spontaneous methods, and one participant was successful at achieving pregnancy spontaneously (34, 59). In total, 9 strips participants had pregnancies that resulted in a live birth. One participant has a total of two separate live births from both ART and spontaneous pregnancies. The live birth rate for the strips groups was 56.3% and there was a total of 12 live births (2 ART, 10 spontaneous) (**Table 4**). Two strips participants birthed twins (34, 61). Two participants in the strips groups had miscarriages, one participant had a termination of pregnancy, and one participant had an ongoing pregnancy at the end of the study (59, 63). In total, four participants in the strips group who attempted pregnancy had a second transplant (34, 63). Two participants were unsuccessful in obtaining pregnancy with the first transplant, so to increase chances of pregnancy they underwent a second OTT (**Table 4**) (34, 63). The first participant achieved three spontaneous pregnancies that resulted in two separate successful live births and one miscarriage (63). The second participant had a successful spontaneous pregnancy and subsequent live birth (34, 63). Of the two other participants in the strips group that had a second OTTs, one participant got pregnant with the first OTT but resulted in a miscarriage, and the other had a termination of pregnancy (63). These two participants had a successful spontaneous pregnancy and live birth with the second transplant (63).

The square group had a pregnancy rate of 45.5%. These participants obtained pregnancy with ART (n=2) or spontaneously (n=3) (**Table 4**) **(** 78, 84, 91–95). In addition, there were a total of five pregnancies in this group; two were achieved using ART, and three were achieved spontaneously. In total, two participants in the squares group had a termination of pregnancy, and one participant had an ongoing pregnancy at the end of the study (78, 92, 95). The live birth rate for the squares group was 18.2%. In this group, 10 participants had a second transplantation and 8 of these participants were not included for pregnancy outcomes due to unclear pregnancy attempts (**Table 4**) (91, 93). A total of 3 spontaneous pregnancies were obtained from these excluded participants (93). Two participants had a total of two separate pregnancies, where in one participant both pregnancies resulted in termination and in the other participant both pregnancies resulted in live birth (93). The third participant that was excluded had a single spontaneous pregnancy which was terminated (93). Two participants were included that underwent a second OTT (**Table 4**) (91, 93). One participant attempted pregnancy spontaneously with first transplantation to increase chances of pregnancy, and then requested a second transplant that resulted in a successful live birth (91). The other participant was unsuccessful at obtaining pregnancy with the first and second OTT (91).

In the fragments group, 3 participants attempted pregnancy spontaneously (**Table 4**) (21, 111, 118). Two of these participants were successful at obtaining pregnancy and live birth (21, 111). In this group, one participant had a total of three separate OTTs (118). This participant attempted pregnancy with the first transplant, however, due to decline ovarian hormone levels the participant had multiple transplants that resulted in a single pregnancy and live birth (118).

### OTT Hormone Restoration and Function Longevity

**Figure 5:**
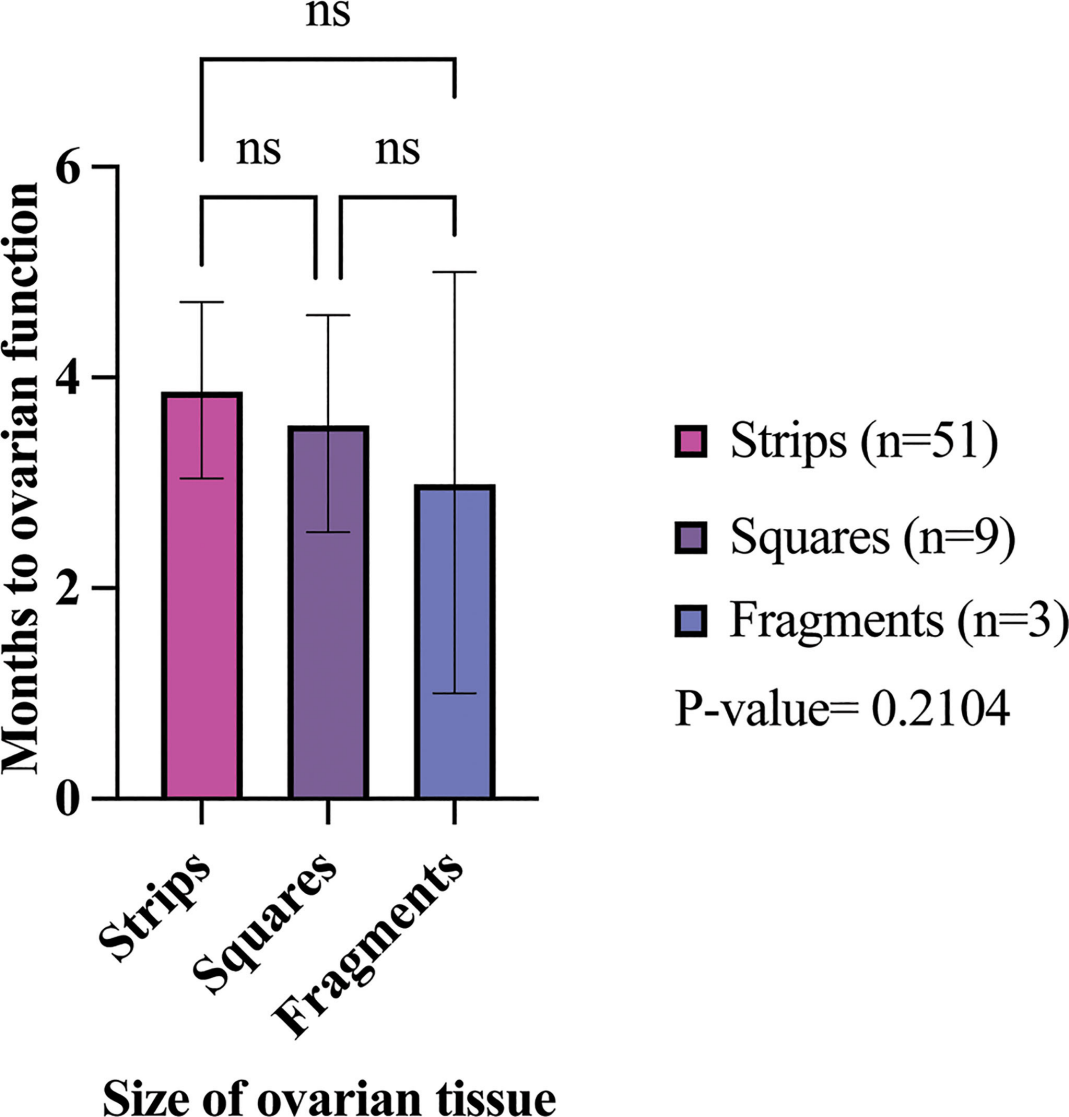
Months to restoration of ovarian activity in different size transplanted ovarian tissues. The average time to ovarian restoration per first OTT was 3.88, 3.56, and 3 months in the strip, square, and fragment groups, respectively. P-values greater than 0.05 were considered not significantly different (ns) (21, 22, 25, 34, 37, 44, 57, 59, 61, 64, 66, 78, 84, 91–93, 95, 111, 115, 118, 124).

Decreased FSH levels or menstrual cycle resumption and the duration of ovarian hormone production post-transplantation determined ovarian function. Overall, 98% of participants had restoration of ovarian function with first OTT; only one participant in the strips group showed no reestablishment of ovarian activity **(** **Table 5** **)** (34). The mean time from OTT to ovarian hormone restoration was 3.88 months (range: 1.6-7.5 months), 3.56 months (range: 1.8-4.6), and 3 months (range:1-5 months) in the strip, square, and fragment groups per first OTT, respectively **(** **Table 5**; **Figure 5** **)** (21, 22, 25, 34, 37, 44, 57, 59, 61, 64, 66, 78, 84, 91, 92, 95, 111, 115, 124). There was no statistically significant difference of the mean time from OTT to ovarian restoration in all three sizes (P-value= 0.2104) **(** **Figure 5** **)**. In all three groups, over 88% of participants had ovarian activity lasting over six months **(** **Table 5** **)**. One participant who had OTT with strips and one with squares had continuous function at 2.4- and 2.8-months post-transplantation, respectively (34, 92). The strips group had the highest percentage of participants with hormone production lasting over one-year post-transplantation with their first OTT (86%) **(** **Table 5** **)**. In total, two participants from the strips group had ovarian activity lasting over five years with their first OTT (63). The strips, squares, and fragments groups had 84, 55.6, and 100% of participants with ongoing ovarian function with first OTT, respectively. In total, eight (16%) and four (44.4%) participants had cessation of ovarian function with first OTT in the strips and square groups, respectively (44, 63, 64).In total, 10 participants underwent a second OTT(8 strips and 2 squares) **(** **Table 5** **) (** 34, 63, 78, 91). In the strips group, 8 participants had an end of ovarian function with the first OTT at a mean of 1.29 years, (range 0.75-3.2 years) post transplantation (34, 63). Additionally, one participant in the strips group that had two OTT did not have restoration of ovarian function with second OTT (63). Participants with ovarian restoration from second OTT, six participants had ongoing function and one participant had an ovarian cessation at one year post second OTT (34, 63). In the squares group, 4 participants had the end of ovarain fuction, two participant had a second OTT **(** **Table 5** **)** (84, 91). Of the two participants that did not have a second OTT, ovarian function ceased at 2 and 3.75 years, respectively (mean=2.9 years) (84, 91). Two participants from the squares group underwent a second OTT due to cessation of ovarian function at 1.25 years and 7 months post first OTT (78, 91).However, the second OTT in one participant did not restore ovarian activity (91). The other participant had ongoing ovarian activity lasting over 1.5 years with second OTT (91).

**Table 5:** Ovarian function outcome after transplantation.

Size of processed ovarian tissue (No. of OTT Participants)	Months from OTT HP mean ± SD, range	HP lasting ≥6 months n, (%)	HP lasting ≥1 year n, (%)	HP lasting ≥ 2 years n, (%)	HP ≥ 5 years n, (%)	Participants with ongoing ovarian function n, (%)	Participants with reported cessation of ovarianfunction n, (%)
**Strips** 1^st^ (n = 51) 2^nd^ (n = 8)	3.88 ± 0.84, 1.6-7.5	49/51, (96) 7/8, (87.5)	45/51, (88.2) 7/8, (87.5)	19/51, (37.3) 4/8, (50)	2/51, (4) 1/8, (12.5)	42/51, (84) 6/8, (75)	9^q^/51, (17.6) 2^q^/8, (25)
**Squares** 1^st^ (n = 9) 2^nd^ (n = 2)	3.56 ± 1.03, 1.8-4.6	8/9, (88.9) 1/2, (50)	4/9, (44.4) 1/2, (50)	3/9, (33.3) 0/2, (0)	0/9, (0) 0/2, (0)	5/9, (55.6) 1/2, (50)	4/9, (44.4) 1^q^/2, (50)
**Fragments** 1^st^ (n = 3)	3 ± 2, 1-5	3/3, (100)	1/3, (33.3)	0/3, (0)	0/3, (0)	3/3, (100)	0/3, (0)

^q^Participant did not have resumption of ovarian activity. HP, hormone production (21, 22, 25, 34, 37, 44, 57, 59, 61, 64, 66, 78, 84, 91, 92, 95, 111, 115, 124).

## Discussion

There have been several recent systematic reviews of OTC and OTT that have identified the different cryoprotectant protocols OTC, fresh and frozen OTT for hormone restoration, ovarian tissue transport prior to OTC, age at OTC, and vitrification versus slow-freezing methods for OTC (13, 125–127).This is the first systematic review that considered the size of ovarian tissue pieces that were processed for OTC and the outcomes of OTT within those size categories. OTT is a technique emerging in the field of reproductive science and has been performed in over 318 patients (128). The ovarian tissue is processed for OTC by thinning the tissue, removing most of the ovarian medulla and isolating the ovarian cortical tissue. The cortex is then cut into pieces to allow for penetration of the cryoprotectant in preparation for cryopreservation (129). In this systematic review, 58 unique sites reported details on the dimensions used for OTC. This analysis identified that ovarian tissue is cut into multiple different sizes. Ten sites located in the U.S., Belgium, Australia, China, Japan, Israel, Germany, Denmark, and Netherlands processed the ovarian tissue in multiple sizes within the same clinical site. There were no correlations found between age at OTC or diagnosis and the size of processed ovarian tissue. There were no indications of a regional preference for ovarian tissue processing size and, furthermore, seven sites cut ovarian tissue into different dimensions within the same size category. These findings highlight that there is no standard size of cryopreserved ovarian tissue. Studies have shown that fragmentation of the ovarian cortex leads to follicle activation (17). This investigation revealed that 36 sites (62% of sites) process ovarian tissue into strips and is the most predominant cryopreserved size. These findings also demonstrate that most sites around the world process the ovarian tissue to have a thickness of 1-2 mm, which coincides with a thickness which has been shown to reduce ice crystal formation and injury, reduce ischemic time, and increase oxygen diffusion once transplanted (130, 131). Transplantation of ovarian tissue has been reported to restore ovarian activity in 95% of transplantations (12, 128). In the results described here, the ovarian function was restored in 98% of participants that underwent OTT.

Fertility clinics in Denmark and Japan utilize fragmentation of ovarian tissue for POI patients to activate dormant follicles and increase fertilization rates (132, 133). Based on this and other evidence from animal models that suggest that disruption of the microenvironment can initiate follicle activation, it was hypothesized that ovarian tissue used in OTT that had been processed into smaller pieces would result in fewer pregnancies and shorter duration of ovarian function, and a shorter time to initiate ovarian function after OTT (16). After transplantation there is up to an 80% loss in ovarian reserve due to factors such as ischemic time (14, 15). The graft size can influence ischemic time, such that smaller graft sizes can have shorter ischemic time than larger graft sizes, leading to earlier ovarian function (15, 134, 135). The resumption of ovarian activity on average did decrease with a smaller processing size (strips 3.88 months, squares 3.56 months, and fragments 3 months). Additionally, the squares group had the highest rate (44.4%) of participants that had a reported cessation of ovarian activity with the first OTT, followed by the strips group (17.6%). Even though more tissue on average was transplanted in the strips than squares group, the strips group had a shorter ovarian tissue lifespan (1.29 years) for the first OTT compared to squares group (2.9 years). While some of these results followed the expected trend, the longevity of ovarian tissue function in the strips group is lower than the reported literature range of 2-5 years due to the lack of follow-ups on participants with OTT in the reviewed reports (128). If further participant follow-up was performed for each study through to cessation of ovarian function, then the longevity of ovarian tissue function would be better understood. While the analysis accounted for the location of the transplant by excluding heterotopic sites, and the reported results only included tissue that was cryopreserved using a slow freezing and not vitrification technique, there are other confounding factors that may contribute to ovarian tissue function after OTT. We also note that there were studies with exciting findings that did not meet our inclusion criteria. For instance, Oktay et al. have implemented the use of extracellular matrix scaffolds, which show promise in extending graft longevity (136).

OTC followed by OTT has resulted in over 140 published live births worldwide and it has been reported to lead to multiple live births from the same OTT procedure (60, 128). This systematic review of reports that contained information on ovarian tissue processing size and OTT outcomes identified an overall pregnancy rate of 81.3%, 45.5%, 66.7% of participants in strips, squares, and fragments groups, respectively. In the strips and fragments groups, the pregnancy rates were higher than the 50% pregnancy rate from OTT reported in the literature (12). This vast difference in pregnancy rate is probably attributed to the fact that authors have not published or described the additional information required for this analysis. The live birth rate was higher in the strips groups (56.3%) compared to squares (18.2%). However, in these two groups, participants (2 squares, 1 strips) had a termination of pregnancy which could have impacted the live birth rate (63, 92, 93, 95). The fragments group had the highest birth rate (66.7%) in all three groups. The small sample size of participants in the fragments group (n=4) compared to the strips (n=51) and squares (n=37) was a contributing factor to these high rates. The participant data utilized for this systematic review was limited by the number of published case studies that contain participants who underwent autologous orthotopic OTT, follow-up time points in studies that discussed OTT outcomes, and contained data on the ovarian tissue sizes. This systematic review relies heavily on the information of case studies, therefore, can introduce an internal validity threat to our reported results. An additional caveat to this study is that authors publishing on OTT might not be reporting unsuccessful OTT cases and have not reported detailed follow-ups of past reported case studies. Evidence is needed to identify and understand factors that could contribute to follicle burnout within the first few months post-OTT, such as impact of tissue fragmentation on preventing ischemia-reperfusion injury and initiating angiogenesis, to develop a unified best practice for ovarian tissue processing (20).

## Conclusion

This systematic review determined the processing sizes of cryopreserved ovarian tissue in sites across the world and examined ovarian function restoration in different sized ovarian tissue pieces in OTT participants. It is shown in this review that there is no standard for processing ovarian tissue and documentation of ovarian restoration outcomes in participants. Although the time of resumption of ovarian activity was not statistically significant between the different sizes, this could have been due to the small population size of the fragments and squares group. The participant population size used in this systematic review could have been more prominent by changing the rigorous inclusion/exclusion criteria to include studies that did not contain enough information about the participants. However, changing the inclusion/exclusion criteria of this systematic review to include studies that lacked important participant information would increase bias within the review. This systematic review has shown the importance of documenting information on the participant before OTC, explicit ovarian tissue processing and transplantation methods, and updating participants’ ovarian outcomes post-transplantation *via* follow-ups. This call to action of proper documentation and consistent OTT participant follow-ups will allow scientists and clinicians to drive research questions with the goals of improving OTC/OTT and maximizing fertility outcomes.

## Data Availability Statement

The original contributions presented in the study are included in the article/**Supplementary Material**. Further inquiries can be directed to the corresponding author.

## Author Contributions

AD and ML contributed to the study concept, design, and data interpretation. AD contributed to data analysis. HK contributed to the study quality assessment and unbiased reviewer. AD, HK, and ML contributed to manuscript preparation. NH, MH, and AD contributed to data collection. All authors contributed to the article and approved the submitted version.

## Funding

This works was supported in part by the Children’s Research Fund, the Ann & Robert H. Lurie Children’s Hospital Faculty Practice Plan award (ML), the Warren and Eloise Batts endowment (ML).

## Conflict of Interest

ML is an Advisor for Dimension Inx, LLC.

The remaining authors declare that the research was conducted in the absence of any commercial or financial relationships that could be construed as a potential conflict of interest.

## Publisher’s Note

All claims expressed in this article are solely those of the authors and do not necessarily represent those of their affiliated organizations, or those of the publisher, the editors and the reviewers. Any product that may be evaluated in this article, or claim that may be made by its manufacturer, is not guaranteed or endorsed by the publisher.
