# Supplementary material for: A Systematic Review of Ovarian Tissue Transplantation Outcomes by Ovarian Tissue Processing Size for Cryopreservation
Source: Front Endocrinol (Lausanne). 2022 Jun 10;13:918899. doi: 10.3389/fendo.2022.918899 (PMC9239173; doi:10.3389/fendo.2022.918899)
Supplement: Supplementary file 1 [file Table_1.docx]

Supplementary Material

## Supplementary Tables

| Author, Year | A | B | C | D | E | F | G | H | Risk |
| --- | --- | --- | --- | --- | --- | --- | --- | --- | --- |
| Schmidt, 2004 | Yes | Yes | Yes | Yes | Yes | Yes | No | Yes | low |
| Donnez, 2004 | Yes | Yes | Yes | Yes | Yes | Yes | Yes | Yes | medium |
| Andersen, 2008 | Yes | No | Yes | Yes | Yes | Yes | No | Yes | medium |
| Greve, 2010 | Yes | Yes | Yes | Yes | Yes | Yes | No | Yes | low |
| Dittrich, 2011 | Yes | Yes | Yes | Yes | Yes | Yes | No | Yes | low |
| Revelli, 2012 | Yes | Yes | Yes | Yes | Yes | Yes | No | Yes | low |
| Ernst, 2013 | Yes | Yes | Yes | Yes | Yes | Yes | No | Yes | low |
| Ernst, 2013 | Yes | Yes | Yes | Yes | Yes | Yes | No | Yes | low |
| Burmeister, 2013 | Yes | Yes | Yes | Yes | Yes | Yes | No | Yes | low |
| Rodriguez-Wallberg, 2015 | Yes | Yes | Yes | Yes | Yes | Yes | No | Yes | low |
| Póvoa, 2016 | Yes | Yes | Yes | Yes | Yes | Yes | No | No | medium |
| Ruan, 2018 | Yes | Yes | Yes | Yes | Yes | Yes | No | Yes | low |
| Chehab, 2019 | Yes | Yes | Yes | Yes | Yes | Yes | No | Yes | low |
| Sonmezer, 2020 | Yes | Yes | Yes | Yes | Yes | Yes | No | Yes | low |
| Hoekman EJ, 2020 | Yes | No | Yes | Yes | Yes | Yes | No | Yes | medium |
| Ruan, 2021 | Yes | Yes | Yes | Yes | Yes | Yes | No | Yes | low |

**Supplementary Table 1.** Risk of bias assessment of Joanna Briggs Institute Critical appraisal for case reports.

1. Were participant’s demographic characteristics clearly described?
2. Was the participant’s history clearly described and presented as a timeline?
3. Was the current clinical condition of the participant on presentation clearly described?
4. Were diagnostic tests or assessment methods and the results clearly described?
5. Was the intervention(s) or treatment procedure(s) clearly described?
6. Was the post-intervention clinical condition clearly described?
7. Were adverse events (harms) or unanticipated events identified and described?
8. Does the case report provide takeaway lessons?

| Author, Year | A | B | C | D | E | F | G | H | I | J | Risk |
| --- | --- | --- | --- | --- | --- | --- | --- | --- | --- | --- | --- |
| Donnez, 2008 | Yes | Yes | Unclear | Unclear | Unclear | Yes | Yes | Yes | No | N/A | low |
| Fabbri, 2014 | Yes | Yes | Yes | Unclear | Unclear | Yes | Yes | Yes | Unclear | N/A | low |
| Poirot, 2019 | Yes | Yes | Unclear | Yes | Yes | Yes | Yes | Yes | No | N/A | low |
| Hjorth, 2020 | Yes | Yes | Yes | Yes | Yes | No | Unclear | Yes | No | N/A | medium |
| Ruan, 2020 | Yes | Yes | Yes | Yes | Unclear | Yes | Yes | Yes | No | N/A | low |

**Supplementary Table 2.** Risk of bias assessment of Joanna Briggs Institute Critical appraisal for case series.

1. Were there clear criteria for inclusion in the case series?
2. Was the condition measured in a standard, reliable way for all participants included in the case series?
3. Were valid methods used for identification of the condition for all participants included in the case series?
4. Did the case series have consecutive inclusion of participants?
5. Did the case series have complete inclusion of participants?
6. Was there clear reporting of the demographics of the participants in the study?
7. Was there clear reporting of clinical information of the participants?
8. Were the outcomes or follow up results of cases clearly reported?
9. Was there clear reporting of the presenting site(s)/clinic(s) demographic information?
10. Was statistical analysis appropriate?

| **Author, Year** | **Country** | **Subgroup** | **Size of tissue cryopreserved**  **based on dimension** |
| --- | --- | --- | --- |
| Gook, 2005 | Australia | OTC | Fragments |
| Seshadri, 2006 | Australia | OTC | Fragments |
| Stern, 2011 | Australia | OTC | Fragments |
| Stern, 2013 | Australia | OTC | Fragments |
| Burmeister, 2013 | Australia | OTC/OTT | Squares |
| Rozen, 2021 | Australia | OTC | Squares |
| Gook, 2021 | Australia | OTC | Strips |
| Ott, 2010 | Austria | OTC | Strips |
| Mayerhofer, 2010 | Austria | OTC | Strips |
| Van der Ven, 2016 | Austria | OTC | Fragments |
| Donnez, 2004 | Belgium | OTC/OTT | Strips, Fragments |
| Demeestere, 2006 | Belgium | OTC | Squares |
| Donnez, 2008 | Belgium | OTC/OTT | Strips, Fragments |
| Dolmans, 2009 | Belgium | OTC | Strips |
| Donnez, 2011 | Belgium | OTC | Fragments |
| Dolmans, 2013 | Belgium | OTC | Strips |
| Imbert, 2014 | Belgium | OTC | Squares |
| Demeestere, 2015 | Belgium | OTC | Fragments |
| Fasano, 2017 | Belgium | OTC | Squares |
| De Roo, 2020 | Belgium | OTC | Squares |
| Delattre, 2020 | Belgium | OTC | Strips |
| Philippart, 2021 | Belgium | OTC | Strips |
| Huang, 2008 | Canada | OTC | Fragments |
| Huang, 2008 | Canada | OTC | Fragments |
| Elizur, 2009 | Canada | OTC | Fragments |
| Ruan, 2018 | China | OTC/OTT | Strips |
| Li, 2019 | China | OTC | Strips |
| Ruan, 2020 | China | OTC/OTT | Strips |
| Du, 2021 | China | OTC | Strips |
| Ruan, 2021 | China | OTC/OTT | Strips |
| Cheng, 2021 | China | OTC/OTT | Fragments |
| Schmidt, 2004 | Denmark | OTC/OTT | Squares |
| Schmidt, 2005 | Denmark | OTC | Squares |
| Rosendahl, 2006 | Denmark | OTC | Squares |
| Andersen, 2008 | Denmark | OTC/OTT | Squares |
| Greve, 2010 | Denmark | OTC/OTT | Squares |
| Greve, 2012 | Denmark | OTC | Squares |
| Greve, 2012 | Denmark | OTC | Squares |
| Ernst, 2012 | Denmark | OTC/OTT | Strips |
| Ernst, 2013 | Denmark | OTC/OTT | Squares |
| El Issaoui, 2016 | Denmark | OTC | Squares |
| Mamsen, 2018 | Denmark | OTC | Fragments, Squares |
| Hjorth, 2020 | Denmark | OTC/OTT | Squares |
| Sermondade, 2018 | France | OTC | Strips |
| Poirot, 2019 | France | OTC | Strips |
| Poirot, 2019 | France | OTC/OTT | Strips |
| Isachenko, 2004 | Germany | OTC | Fragments |
| Maltaris, 2006 | Germany | OTC | Fragments |
| Maltaris, 2006 | Germany | OTC | Fragments |
| Maltaris, 2007 | Germany | OTC | Fragments |
| Dittrich, 2008 | Germany | OTC | Fragments |
| Dittrich, 2012 | Germany | OTC/OTT | Fragments |
| Lotz, 2014 | Germany | OTC | Fragments |
| Dittrich, 2015 | Germany | OTC | Fragments |
| Van der Ven, 2016 | Germany, Austria, Switzerland | OTC | Strips, Fragments |
| Raffel, 2017 | Germany | OTC | Fragments |
| Chehab, 2019 | Germany | OTC/OTT | Strips |
| Liebenthron, 2019 | Germany | OTC | Strips |
| Abtahi, 2016 | Iran | OTC | Strips |
| Mohsenzadeh, 2017 | Iran | OTC | Strips |
| Meirow, 2007 | Israel | OTC | Strips |
| Meirow, 2008 | Israel | OTC | Strips |
| Azem, 2009 | Israel | OTC | Strips |
| Meirow, 2016 | Israel | OTC | Strips |
| Abir, 2016 | Israel | OTC | Strips |
| Karavani, 2018 | Israel | OTC | Strips |
| Karavani, 2019 | Israel | OTC | Strips |
| Fouks, 2020 | Israel | OTC | Fragments |
| Shai, 2021 | Israel | OTC | Strips |
| Fabbri, 2012 | Italy | OTC | Strips |
| Revelli, 2013 | Italy | OTC/OTT | Squares |
| Fabbri, 2014 | Italy | OTC/OTT | Strips |
| Kikuchi, 2013 | Japan | OTC | Strips |
| Takae, 2015 | Japan | OTC | Squares |
| Kawamura, 2020 | Japan | OTC | Squares |
| Meng, 2021 | Japan | OTC | Squares |
| Takae, 2021 | Japan | OTC | Strips, Squares |
| Lee, 2018 | Korea | OTC | Squares |
| Jenninga, 2008 | Netherlands | OTC | Strips |
| Janse, 2011 | Netherlands | OTC | Strips, Fragments |
| Jenninga, 2012 | Netherlands | OTC | Strips |
| Bastings, 2014 | Netherlands | OTC | Strips |
| Hoekman, 2015 | Netherlands | OTC | Strips |
| Peek, 2019 | Netherlands | OTC | Strips |
| Hoekman, 2020 | Netherlands | OTC/OTT | Strips |
| Tanbo, 2015 | Norway | OTC | Squares |
| Radwan, 2016 | Poland | OTC | Squares |
| Póvoa,2016 | Portugal | OTC/OTT | Fragments |
| Bystrova, 2019 | Russia | OTC | Squares |
| Virant-Klun, 2018 | Slovenia | OTC | Strips |
| Kim, 2004 | South Korea | OTC | Squares |
| González, 2011 | Spain | OTC | Strips |
| Policiano, 2020 | Spain | OTC | Strips |
| Rodriguez-Wallberg, 2015 | Sweden | OTC/OTT | Fragments |
| Sonmezer, 2020 | Turkey | OTC/OTT | Strips |
| Radford, 2001 | UK | OTC | Strips |
| Walker, 2021 | UK | OTC | Fragments |
| Oktay, 2008 | USA | OTC | Strips |
| Gracia, 2012 | USA | OTC | Strips |
| Kim, 2012 | USA | OTC | Squares |
| Hickman, 2015 | USA | OTC | Strips |
| Silber, 2018 | USA | OTC | Strips, Squares |
| Hanfling, 2021 | USA | OTC | Strips |
| Schermerhorn, 2021 | USA | OTC | Strips |

**Supplemental Table 3.** Summary of articles used for OTC processing analysis.

(1-53)((2, 3, 44, 47, 54-104)

| **Author,**  **Year** | **Country** | **Subgroup** | **No. of OTT Participants** | **Size of tissue cryopreserved** | **Outcome(s)** | **Notes** |
| --- | --- | --- | --- | --- | --- | --- |
| Schmidt, 2004 | Denmark | OTC/OTT | 1 | Squares | Failed ICSI fertilization due to poor semen quality | Participant had ongoing ovarian activity at the end of study |
| Donnez, 2004/ 2008 | Belgium | OTC/OTT | 5 | Strips, fragments | 1 Live Birth | Only 1 participant was included to information on amount of tissue transplanted. This participant was described in two reports (2004 and 2008). This participant had tissue processed into strips and fragments. Fragments were transplanted. |
| Andersen, 2008 | Denmark | OTC/OTT | 6 | Squares | 2 live births, 1 miscarriage | 2 out of 6 participants which had ortho/heterotopic transplant were excluded. Of the participants that were included, 2 participants had 2 orthotopic transplants |
| Greve, 2010 | Denmark | OTC/OTT | 1 | Squares | 1 Pregnancy | Participant had to terminate pregnancy |
| Dittrich, 2012 | Germany | OTC/OTT | 1 | Fragments | 1 Live birth | Participant had ongoing ovarian activity at the end of study |
| Ernst, 2012 | Denmark | OTC/OTT | 1 | Strips | Had cession of ovarian tissue after 19 months |  |
| Ernst, 2013 | Denmark | OTC/OTT | 1 | Squares | 1 Pregnancy | Had to terminate pregnancy due to reoccurrence of cancer, was included as 1 pregnancy |
| Burmeister, 2013 | Australia | OTC/OTT | 1 | Squares | 1 Pregnancy | Had 1 ongoing pregnancy at the end of the study |
| Revelli, 2013 | Italy | OTC/OTT | 1 | Squares | 1 Live birth | Participant had ongoing ovarian activity at the end of study |
| Fabbri, 2014 | Italy | OTC/OTT | 3 | Strips | Ovarian restoration | 1 Participant was excluded due to heterotopic transplantation |
| Rodriguez-Wallberg, 2015 | Sweden | OTC/OTT | 1 | Fragments | 1 Live birth | Participant had three orthotopic transplants due to declining levels in ovarian hormones, after 6 IVF cycles resulted in 3 good quality embryos, one which was transplanted resulted in live birth |
| Póvoa, 2016 | Portugal | OTC/OTT | 1 | Fragments | Ovarian restoration | Participant continued to have ovarian restoration 7 months after transplantation |
| Ruan, 2018 | China | OTC/OTT | 1 | Strips | Ovarian function restoration | Participant had ongoing ovarian activity at the end of study |
| Poirot, 2019 | France | OTC/OTT | 31 | Strips | 8 live births, 3 miscarriages, 1 abortion | 4 out of the 31 participants that underwent ortho-heterotopic were excluded |
| Chehab, 2019 | Germany | OTC/OTT | 1 | Strips | 2 Live births | Participant had ongoing ovarian activity at the end of study |
| Hoekman, 2020 | Netherlands | OTC/OTT | 67 | Strips | 6 Live births | 7 participants out of 67 underwent transplantation, 1 participant had to terminate pregnancy |
| Hjorth, 2020 | Denmark | OTC/OTT | 28 | Squares | 5 participants had live births; 9 participants had termination of pregnancy | These participants were only included for fertility outcomes |
| Sonmezer, 2020 | Turkey | OTC/OTT | 1 | Strips | 1 Live birth | Participant had ongoing ovarian activity at the end of study |
| Ruan, 2020 | China | OTC/OTT | 10 | Strips | Ovarian function restoration | All participants had ongoing ovarian activity at the end of study |
| Ruan, 2021 | China | OTC/OTT | 1 | Strips | Ovarian function restoration | Participant had ongoing ovarian activity at the end of study |

**Supplementary Table 4.** Summary of articles used of OTT outcomes analysis.

(4, 7, 16, 19, 26, 39, 41, 43, 46, 48, 60, 66, 73-75, 77, 93, 97, 100, 105, 106)

**References**

1. Meirow D, Ra'anani H, Shapira M, Brenghausen M, Derech Chaim S, Aviel-Ronen S, et al. Transplantations of frozen-thawed ovarian tissue demonstrate high reproductive performance and the need to revise restrictive criteria. Fertil Steril. 2016;106(2):467-74.

2. Silber SJ, DeRosa M, Goldsmith S, Fan Y, Castleman L, Melnick J. Cryopreservation and transplantation of ovarian tissue: results from one center in the USA. J Assist Reprod Genet. 2018;35(12):2205-13.

3. Donnez J, Dolmans MM, Demylle D, Jadoul P, Pirard C, Squifflet J, et al. Livebirth after orthotopic transplantation of cryopreserved ovarian tissue. Lancet. 2004;364(9443):1405-10.

4. Donnez J, Squifflet J, Van Eyck AS, Demylle D, Jadoul P, Van Langendonckt A, et al. Restoration of ovarian function in orthotopically transplanted cryopreserved ovarian tissue: a pilot experience. Reprod Biomed Online. 2008;16(5):694-704.

5. Mayerhofer K, Ott J, Nouri K, Stoegbauer L, Fischer EM, Lipovac M, et al. Laparoscopic ovarian tissue harvesting for cryopreservation: an effective and safe procedure for fertility preservation. Eur J Obstet Gynecol Reprod Biol. 2010;152(1):68-72.

6. Ott J, Nouri K, Stögbauer L, Fischer EM, Lipovac M, Promberger R, et al. Ovarian tissue cryopreservation for non-malignant indications. Arch Gynecol Obstet. 2010;281(4):735-9.

7. Ruan X, Du J, Korell M, Kong W, Lu D, Jin F, et al. Case report of the first successful cryopreserved ovarian tissue retransplantation in China. Climacteric. 2018;21(6):613-6.

8. González C, Devesa M, Boada M, Coroleu B, Veiga A, Barri PN. Combined strategy for fertility preservation in an oncologic patient: vitrification of in vitro matured oocytes and ovarian tissue freezing. J Assist Reprod Genet. 2011;28(12):1147-9.

9. Virant-Klun I, Vogler A. In vitro maturation of oocytes from excised ovarian tissue in a patient with autoimmune ovarian insufficiency possibly associated with Epstein-Barr virus infection. Reprod Biol Endocrinol. 2018;16(1):33.

10. Philippart C, Masciangelo R, Camboni A, Donnez J, Dolmans MM. Basal lamina characterization in frozen-thawed and long-term grafted human prepubertal ovarian tissue. Reprod Biomed Online. 2021;42(5):859-69.

11. Dolmans MM, Jadoul P, Gilliaux S, Amorim CA, Luyckx V, Squifflet J, et al. A review of 15 years of ovarian tissue bank activities. J Assist Reprod Genet. 2013;30(3):305-14.

12. Abir R, Ben-Aharon I, Garor R, Yaniv I, Ash S, Stemmer SM, et al. Cryopreservation of in vitro matured oocytes in addition to ovarian tissue freezing for fertility preservation in paediatric female cancer patients before and after cancer therapy. Hum Reprod. 2016;31(4):750-62.

13. Abtahi NS, Ebrahimi B, Fathi R, Khodaverdi S, Mehdizadeh Kashi A, Valojerdi MR. An Introduction to The Royan Human Ovarian Tissue Bank. Int J Fertil Steril. 2016;10(2):261-3.

14. Jenninga E, Louwe LA, Peters AA, Nortier JW, Hilders CG. Timing of fertility preservation procedures in a cohort of female patients with cancer. Eur J Obstet Gynecol Reprod Biol. 2012;160(2):170-3.

15. Hoekman EJ, Smit VT, Fleming TP, Louwe LA, Fleuren GJ, Hilders CG. Searching for metastases in ovarian tissue before autotransplantation: a tailor-made approach. Fertil Steril. 2015;103(2):469-77.

16. Hoekman EJ, Louwe LA, Rooijers M, Westerlaken LAJ, Klijn NF, Pilgram GSK, et al. Ovarian tissue cryopreservation: Low usage rates and high live‐birth rate after transplantation. Acta Obstetricia et Gynecologica Scandinavica. 2020;99(2):213-21.

17. Jenninga E, Hilders CG, Louwe LA, Peters AA. Female fertility preservation: practical and ethical considerations of an underused procedure. Cancer J. 2008;14(5):333-9.

18. Bastings L, Liebenthron J, Westphal JR, Beerendonk CC, van der Ven H, Meinecke B, et al. Efficacy of ovarian tissue cryopreservation in a major European center. J Assist Reprod Genet. 2014;31(8):1003-12.

19. Sonmezer M, Ozkavukcu S, Sukur YE, Kankaya D, Arslan O. First pregnancy and live birth in Turkey following frozen-thawed ovarian tissue transplantation in a patient with acute lymphoblastic leukemia who underwent cord blood transplantation. J Assist Reprod Genet. 2020;37(8):2033-43.

20. Radford JA, Lieberman BA, Brison DR, Smith AR, Critchlow JD, Russell SA, et al. Orthotopic reimplantation of cryopreserved ovarian cortical strips after high-dose chemotherapy for Hodgkin's lymphoma. Lancet. 2001;357(9263):1172-5.

21. Gracia CR, Chang J, Kondapalli L, Prewitt M, Carlson CA, Mattei P, et al. Ovarian tissue cryopreservation for fertility preservation in cancer patients: successful establishment and feasibility of a multidisciplinary collaboration. J Assist Reprod Genet. 2012;29(6):495-502.

22. Hanfling SN, Parikh T, Mayhew A, Robinson E, Graham J, Gomez-Lobo V, et al. Case report: two cases of mature oocytes found in prepubertal girls during ovarian tissue cryopreservation. F S Rep. 2021;2(3):296-9.

23. Sermondade N, Sonigo C, Sifer C, Valtat S, Ziol M, Eustache F, et al. Serum antimüllerian hormone is associated with the number of oocytes matured in vitro and with primordial follicle density in candidates for fertility preservation. Fertil Steril. 2019;111(2):357-62.

24. Shai D, Aviel-Ronen S, Spector I, Raanani H, Shapira M, Gat I, et al. Ovaries of patients recently treated with alkylating agent chemotherapy indicate the presence of acute follicle activation, elucidating its role among other proposed mechanisms of follicle loss. Fertil Steril. 2021;115(5):1239-49.

25. Azem F, Hasson J, Cohen T, Shwartz T, Mey-Raz N, Almog B, et al. Retrieval of immature oocytes after chemotherapy for Hodgkin's disease and prolonged ovarian down-regulation with gonadotropin-releasing hormone agonist. Fertil Steril. 2009;92(2):828.e1-2.

26. Fabbri R, Pasquinelli G, Magnani V, Macciocca M, Vicenti R, Parazza I, et al. Autotransplantation of cryopreserved ovarian tissue in oncological patients: recovery of ovarian function. Future Oncol. 2014;10(4):549-61.

27. Meirow D, Baum M, Yaron R, Levron J, Hardan I, Schiff E, et al. Ovarian tissue cryopreservation in hematologic malignancy: ten years' experience. Leuk Lymphoma. 2007;48(8):1569-76.

28. Meirow D, Hardan I, Dor J, Fridman E, Elizur S, Ra'anani H, et al. Searching for evidence of disease and malignant cell contamination in ovarian tissue stored from hematologic cancer patients. Hum Reprod. 2008;23(5):1007-13.

29. Hickman LC, Uy-Kroh MJ, Chiesa-Vottero A, Desai N, Flyckt R. Ovarian Tissue Cryopreservation for Benign Gynecologic Disease: A Case of Ovarian Torsion and Review of the Literature. J Minim Invasive Gynecol. 2016;23(3):446-9.

30. Oktay K, Oktem O. Ovarian cryopreservation and transplantation for fertility preservation for medical indications: report of an ongoing experience. Fertil Steril. 2010;93(3):762-8.

31. Schermerhorn SMV, Rosen MP, Blevins EM, Byrd KA, Rabban JT, Marsh P, et al. Regional air transportation of ovarian tissue for cryopreservation in a prepubertal female with cancer. Pediatr Blood Cancer. 2021;68(9):e29107.

32. Dolmans MM, Donnez J, Camboni A, Demylle D, Amorim C, Van Langendonckt A, et al. IVF outcome in patients with orthotopically transplanted ovarian tissue. Hum Reprod. 2009;24(11):2778-87.

33. Fabbri R, Vicenti R, Macciocca M, Pasquinelli G, Lima M, Parazza I, et al. Cryopreservation of ovarian tissue in pediatric patients. Obstet Gynecol Int. 2012;2012:910698.

34. Janse F, Donnez J, Anckaert E, De Jong FH, Fauser BCJM, Dolmans M-M. Limited Value of Ovarian Function Markers following Orthotopic Transplantation of Ovarian Tissue after Gonadotoxic Treatment. 2011;96(4):1136-44.

35. Policiano C, Subirá J, Aguilar A, Monzó S, Iniesta I, Rubio Rubio JM. Impact of ABVD chemotherapy on ovarian reserve after fertility preservation in reproductive-aged women with Hodgkin lymphoma. J Assist Reprod Genet. 2020;37(7):1755-61.

36. Delattre S, Segers I, Van Moer E, Drakopoulos P, Mateizel I, Enghels L, et al. Combining fertility preservation procedures to spread the eggs across different baskets: a feasibility study. Hum Reprod. 2020;35(11):2524-36.

37. Peek R, Schleedoorn M, Smeets D, van de Zande G, Groenman F, Braat D, et al. Ovarian follicles of young patients with Turner's syndrome contain normal oocytes but monosomic 45,X granulosa cells. Hum Reprod. 2019;34(9):1686-96.

38. Li Y, Ruan X, Liebenthron J, Montag M, Zhou Q, Kong W, et al. Ovarian tissue cryopreservation for patients with premature ovary insufficiency caused by cancer treatment: optimal protocol. Climacteric. 2019;22(4):383-9.

39. Ruan X, Cheng J, Korell M, Du J, Kong W, Lu D, et al. Ovarian tissue cryopreservation and transplantation prevents iatrogenic premature ovarian insufficiency: first 10 cases in China. Climacteric. 2020;23(6):574-80.

40. Du J, Ruan X, Jin F, Li Y, Cheng J, Gu M, et al. Abnormalities of early folliculogenesis and serum anti-Müllerian hormone in chinese patients with polycystic ovary syndrome. J Ovarian Res. 2021;14(1):36.

41. Ruan X, Du J, Lu D, Duan W, Jin F, Kong W, et al. First pregnancy in China after ovarian tissue transplantation to prevent premature ovarian insufficiency. Climacteric. 2021;24(6):624-8.

42. Liebenthron J, Montag M, Reinsberg J, Köster M, Isachenko V, van der Ven K, et al. Overnight ovarian tissue transportation for centralized cryobanking: a feasible option. Reprod Biomed Online. 2019;38(5):740-9.

43. Chehab G, Krüssel J, Fehm T, Fischer-Betz R, Schneider M, Germeyer A, et al. Successful conception in a 34-year-old lupus patient following spontaneous pregnancy after autotransplantation of cryopreserved ovarian tissue. Lupus. 2019;28(5):675-80.

44. Van der Ven H, Liebenthron J, Beckmann M, Toth B, Korell M, Krüssel J, et al. Ninety-five orthotopic transplantations in 74 women of ovarian tissue after cytotoxic treatment in a fertility preservation network: tissue activity, pregnancy and delivery rates. Hum Reprod. 2016;31(9):2031-41.

45. Poirot C, Fortin A, Lacorte JM, Akakpo JP, Genestie C, Vernant JP, et al. Impact of cancer chemotherapy before ovarian cortex cryopreservation on ovarian tissue transplantation. Hum Reprod. 2019;34(6):1083-94.

46. Ernst E, Kjærsgaard M, Birkebæk NH, Clausen N, Andersen CY. Case report: stimulation of puberty in a girl with chemo- and radiation therapy induced ovarian failure by transplantation of a small part of her frozen/thawed ovarian tissue. Eur J Cancer. 2013;49(4):911-4.

47. Takae S, Furuta S, Keino D, Shiraishi E, Iwahata Y, Oyama K, et al. Surgical management of unilateral oophorectomy for ovarian tissue cryopreservation in high-risk children and adolescents with varied backgrounds. Pediatr Surg Int. 2021;37(8):1021-9.

48. Poirot C, Brugieres L, Yakouben K, Prades-Borio M, Marzouk F, de Lambert G, et al. Ovarian tissue cryopreservation for fertility preservation in 418 girls and adolescents up to 15 years of age facing highly gonadotoxic treatment. Twenty years of experience at a single center. Acta Obstet Gynecol Scand. 2019;98(5):630-7.

49. Mohsenzadeh M, Khalili MA, Tabibnejad N, Yari N, Agha-Rahimi A, Karimi-Zarchi M. Embryo Cryopreservation Following In-Vitro Maturation for Fertility Preservation in a Woman with Mullerian Adenosarcoma: A Case Report. J Hum Reprod Sci. 102017. p. 138-41.

50. Gook D, Hale L, Polyakov A, Manley T, Rozen G, Stern K. Experience with transplantation of human cryopreserved ovarian tissue to a sub-peritoneal abdominal site. Hum Reprod. 2021;36(9):2473-83.

51. Karavani G, Schachter-Safrai N, Chill HH, Mordechai Daniel T, Bauman D, Revel A. Single-Incision Laparoscopic Surgery for Ovarian Tissue Cryopreservation. J Minim Invasive Gynecol. 2018;25(3):474-9.

52. Karavani G, Schachter-Safrai N, Revel A, Mordechai-Daniel T, Bauman D, Imbar T. In vitro maturation rates in young premenarche patients. Fertil Steril. 2019;112(2):315-22.

53. Greve T, Clasen-Linde E, Andersen MT, Andersen MK, Sørensen SD, Rosendahl M, et al. Cryopreserved ovarian cortex from patients with leukemia in complete remission contains no apparent viable malignant cells. Blood. 2012;120(22):4311-6.

54. Kikuchi I, Kagawa N, Silber S, Kuwayama M, Takehara Y, Aono F, et al. Oophorectomy for fertility preservation via reduced-port laparoscopic surgery. Surg Innov. 2013;20(3):219-24.

55. Takae S, Sugishita Y, Yoshioka N, Hoshina M, Horage Y, Sato Y, et al. The role of menstrual cycle phase and AMH levels in breast cancer patients whose ovarian tissue was cryopreserved for oncofertility treatment. J Assist Reprod Genet. 2015;32(2):305-12.

56. Kawamura K, Ishizuka B, Hsueh AJW. Drug-free in-vitro activation of follicles for infertility treatment in poor ovarian response patients with decreased ovarian reserve. Reprod Biomed Online. 2020;40(2):245-53.

57. Meng L, Kawamura K, Yoshioka N, Tamura M, Furuyama S, Nakajima M, et al. Learning Curve of Surgeons Performing Laparoscopic Ovarian Tissue Transplantation in Women with Premature Ovarian Insufficiency: A Statistical Process Control Analysis. J Minim Invasive Gynecol. 2022;29(4):559-66.

58. Rosendahl M, Loft A, Byskov AG, Ziebe S, Schmidt KT, Andersen AN, et al. Biochemical pregnancy after fertilization of an oocyte aspirated from a heterotopic autotransplant of cryopreserved ovarian tissue: case report. Hum Reprod. 2006;21(8):2006-9.

59. Mamsen LS, Kelsey TW, Ernst E, Macklon KT, Lund AM, Andersen CY. Cryopreservation of ovarian tissue may be considered in young girls with galactosemia. J Assist Reprod Genet. 2018;35(7):1209-17.

60. Burmeister L, Kovacs GT, Osianlis T. First Australian pregnancy after ovarian tissue cryopreservation and subsequent autotransplantation. Med J Aust. 2013;198(3):158-9.

61. Rozen G, Avagliano S, Agresta F, Gook D, Polyakov A, Stern C. Ovarian tissue grafting: Lessons learnt from our experience with 55 grafts. Reprod Med Biol. 2021;20(3):277-88.

62. De Roo C, Lierman S, Tilleman K, De Sutter P. In-vitro fragmentation of ovarian tissue activates primordial follicles through the Hippo pathway. Hum Reprod Open. 2020;2020(4):hoaa048.

63. El Issaoui M, Giorgione V, Mamsen LS, Rechnitzer C, Birkebæk N, Clausen N, et al. Effect of first line cancer treatment on the ovarian reserve and follicular density in girls under the age of 18 years. Fertil Steril. 2016;106(7):1757-62.e1.

64. Greve T, Schmidt KT, Kristensen SG, Ernst E, Andersen CY. Evaluation of the ovarian reserve in women transplanted with frozen and thawed ovarian cortical tissue. Fertil Steril. 2012;97(6):1394-8.e1.

65. Schmidt KL, Andersen CY, Loft A, Byskov AG, Ernst E, Andersen AN. Follow-up of ovarian function post-chemotherapy following ovarian cryopreservation and transplantation. Hum Reprod. 2005;20(12):3539-46.

66. Revelli A, Marchino G, Dolfin E, Molinari E, Delle Piane L, Salvagno F, et al. Live birth after orthotopic grafting of autologous cryopreserved ovarian tissue and spontaneous conception in Italy. Fertil Steril. 2013;99(1):227-30.

67. Lee JR, Lee D, Park S, Paik EC, Kim SK, Jee BC, et al. Successful in Vitro Fertilization and Embryo Transfer after Transplantation of Cryopreserved Ovarian Tissue: Report of the First Korean Case. J Korean Med Sci. 2018;33(21):e156.

68. Tanbo T, Greggains G, Storeng R, Busund B, Langebrekke A, Fedorcsak P. Autotransplantation of cryopreserved ovarian tissue after treatment for malignant disease - the first Norwegian results. Acta Obstet Gynecol Scand. 2015;94(9):937-41.

69. Bystrova O, Lapina E, Kalugina A, Lisyanskaya A, Tapilskaya N, Manikhas G. Heterotopic transplantation of cryopreserved ovarian tissue in cancer patients: a case series. Gynecol Endocrinol. 2019;35(12):1043-9.

70. Kim SS. Assessment of long term endocrine function after transplantation of frozen-thawed human ovarian tissue to the heterotopic site: 10 year longitudinal follow-up study. J Assist Reprod Genet. 2012;29(6):489-93.

71. Imbert R, Moffa F, Tsepelidis S, Simon P, Delbaere A, Devreker F, et al. Safety and usefulness of cryopreservation of ovarian tissue to preserve fertility: a 12-year retrospective analysis. Hum Reprod. 2014;29(9):1931-40.

72. Fasano G, Dechène J, Antonacci R, Biramane J, Vannin AS, Van Langendonckt A, et al. Outcomes of immature oocytes collected from ovarian tissue for cryopreservation in adult and prepubertal patients. Reprod Biomed Online. 2017;34(6):575-82.

73. Andersen CY, Rosendahl M, Byskov AG, Loft A, Ottosen C, Dueholm M, et al. Two successful pregnancies following autotransplantation of frozen/thawed ovarian tissue. Hum Reprod. 2008;23(10):2266-72.

74. Greve T, Ernst E, Markholt S, Schmidt KT, Andersen CY. Legal termination of a pregnancy resulting from transplanted cryopreserved ovarian tissue. Acta Obstet Gynecol Scand. 2010;89(12):1589-91.

75. Dueholm Hjorth IM, Kristensen SG, Dueholm M, Humaidan P. Reproductive outcomes after in vitro fertilization treatment in a cohort of Danish women transplanted with cryopreserved ovarian tissue. Fertil Steril. 2020;114(2):379-87.

76. Tryde Schmidt KL, Yding Andersen C, Starup J, Loft A, Byskov AG, Nyboe Andersen A. Orthotopic autotransplantation of cryopreserved ovarian tissue to a woman cured of cancer - follicular growth, steroid production and oocyte retrieval. Reprod Biomed Online. 2004;8(4):448-53.

77. Ernst EH, Offersen BV, Andersen CY, Ernst E. Legal termination of a pregnancy resulting from transplanted cryopreserved ovarian tissue due to cancer recurrence. J Assist Reprod Genet. 2013;30(7):975-8.

78. Kim SS, Hwang IT, Lee HC. Heterotopic autotransplantation of cryobanked human ovarian tissue as a strategy to restore ovarian function. Fertil Steril. 2004;82(4):930-2.

79. Radwan P, Abramik A, Wilczyński J, Radwan M. Successful autotransplantation of cryopreserved ovarian tissue with recovery of the ovarian function. Ginekol Pol. 2016;87(3):235-40.

80. Demeestere I, Simon P, Buxant F, Robin V, Fernandez SA, Centner J, et al. Ovarian function and spontaneous pregnancy after combined heterotopic and orthotopic cryopreserved ovarian tissue transplantation in a patient previously treated with bone marrow transplantation: case report. Hum Reprod. 2006;21(8):2010-4.

81. Donnez J, Dolmans MM, Demylle D, Jadoul P, Pirard C, Squifflet J, et al. Restoration of ovarian function after orthotopic (intraovarian and periovarian) transplantation of cryopreserved ovarian tissue in a woman treated by bone marrow transplantation for sickle cell anaemia: case report. Hum Reprod. 2006;21(1):183-8.

82. Demeestere I, Simon P, Dedeken L, Moffa F, Tsépélidis S, Brachet C, et al. Live birth after autograft of ovarian tissue cryopreserved during childhood. Hum Reprod. 2015;30(9):2107-9.

83. Donnez J, Squifflet J, Jadoul P, Demylle D, Cheron AC, Van Langendonckt A, et al. Pregnancy and live birth after autotransplantation of frozen-thawed ovarian tissue in a patient with metastatic disease undergoing chemotherapy and hematopoietic stem cell transplantation. Fertil Steril. 2011;95(5):1787.e1-4.

84. Huang JY, Tulandi T, Holzer H, Tan SL, Chian RC. Combining ovarian tissue cryobanking with retrieval of immature oocytes followed by in vitro maturation and vitrification: an additional strategy of fertility preservation. Fertil Steril. 2008;89(3):567-72.

85. Huang JY, Tulandi T, Holzer H, Lau NM, Macdonald S, Tan SL, et al. Cryopreservation of ovarian tissue and in vitro matured oocytes in a female with mosaic Turner syndrome: Case Report. Hum Reprod. 2008;23(2):336-9.

86. Elizur SE, Tulandi T, Meterissian S, Huang JY, Levin D, Tan SL. Fertility preservation for young women with rectal cancer--a combined approach from one referral center. J Gastrointest Surg. 2009;13(6):1111-5.

87. Cheng J, Ruan X, Zhou Q, Li Y, Du J, Jin F, et al. Long-time low-temperature transportation of human ovarian tissue before cryopreservation. Reprod Biomed Online. 2021;43(2):172-83.

88. Isachenko E, Rahimi G, Isachenko V, Nawroth F. In-vitro maturation of germinal-vesicle oocytes and cryopreservation in metaphase I/II: a possible additional option to preserve fertility during ovarian tissue cryopreservation. Reprod Biomed Online. 2004;8(5):553-7.

89. Maltaris T, Koelbl H, Fischl F, Seufert R, Schmidt M, Kohl J, et al. Xenotransplantation of human ovarian tissue pieces in gonadotropin-stimulated SCID mice: the effect of ovariectomy. Anticancer Res. 2006;26(6b):4171-6.

90. Maltaris T, Dragonas C, Hoffmann I, Mueller A, Beckmann MW, Dittrich R. Simple prediction of the survival of follicles in cryopreserved human ovarian tissue. J Reprod Dev. 2006;52(4):577-82.

91. Maltaris T, Beckmann MW, Binder H, Mueller A, Hoffmann I, Koelbl H, et al. The effect of a GnRH agonist on cryopreserved human ovarian grafts in severe combined immunodeficient mice. Reproduction. 2007;133(2):503-9.

92. Dittrich R, Mueller A, Binder H, Oppelt PG, Renner SP, Goecke T, et al. First retransplantation of cryopreserved ovarian tissue following cancer therapy in Germany. Dtsch Arztebl Int. 2008;105(15):274-8.

93. Dittrich R, Lotz L, Keck G, Hoffmann I, Mueller A, Beckmann MW, et al. Live birth after ovarian tissue autotransplantation following overnight transportation before cryopreservation. Fertil Steril. 2012;97(2):387-90.

94. Lotz L, Liebenthron J, Nichols-Burns SM, Montag M, Hoffmann I, Beckmann MW, et al. Spontaneous antral follicle formation and metaphase II oocyte from a non-stimulated prepubertal ovarian tissue xenotransplant. Reprod Biol Endocrinol. 2014;12:41.

95. Dittrich R, Hackl J, Lotz L, Hoffmann I, Beckmann MW. Pregnancies and live births after 20 transplantations of cryopreserved ovarian tissue in a single center. Fertil Steril. 2015;103(2):462-8.

96. Raffel N, Lotz L, Hoffmann I, Liebenthron J, Söder S, Beckmann MW, et al. Repetitive Maturation of Oocytes From Non-Stimulated Xenografted Ovarian Tissue From a Prepubertal Patient Indicating the Independence of Human Ovarian Tissue. Geburtshilfe Frauenheilkd. 2017;77(12):1304-11.

97. Póvoa A, Xavier P, Calejo L, Soares S, Sousa M, Silva J, et al. First transplantation of cryopreserved ovarian tissue in Portugal, stored for 10 years: an unexpected indication. Reprod Biomed Online. 2016;32(3):334-6.

98. Fouks Y, Hamilton E, Cohen Y, Hasson J, Kalma Y, Azem F. In-vitro maturation of oocytes recovered during cryopreservation of pre-pubertal girls undergoing fertility preservation. Reprod Biomed Online. 2020;41(5):869-73.

99. Walker CA, Bjarkadottir BD, Fatum M, Lane S, Williams SA. Variation in follicle health and development in cultured cryopreserved ovarian cortical tissue: a study of ovarian tissue from patients undergoing fertility preservation. Hum Fertil (Camb). 2021;24(3):188-98.

100. Rodriguez-Wallberg KA, Karlström PO, Rezapour M, Castellanos E, Hreinsson J, Rasmussen C, et al. Full-term newborn after repeated ovarian tissue transplants in a patient treated for Ewing sarcoma by sterilizing pelvic irradiation and chemotherapy. Acta Obstet Gynecol Scand. 2015;94(3):324-8.

101. Stern CJ, Gook D, Hale LG, Agresta F, Oldham J, Rozen G, et al. First reported clinical pregnancy following heterotopic grafting of cryopreserved ovarian tissue in a woman after a bilateral oophorectomy. Hum Reprod. 2013;28(11):2996-9.

102. Gook DA, Edgar DH, Borg J, Archer J, McBain JC. Diagnostic assessment of the developmental potential of human cryopreserved ovarian tissue from multiple patients using xenografting. Human Reproduction. 2005;20(1):72-8.

103. Seshadri T, Gook D, Lade S, Spencer A, Grigg A, Tiedemann K, et al. Lack of evidence of disease contamination in ovarian tissue harvested for cryopreservation from patients with Hodgkin lymphoma and analysis of factors predictive of oocyte yield. Br J Cancer. 2006;94(7):1007-10.

104. Stern CJ, Toledo MG, Hale LG, Gook DA, Edgar DH. The first Australian experience of heterotopic grafting of cryopreserved ovarian tissue: evidence of establishment of normal ovarian function. Aust N Z J Obstet Gynaecol. 2011;51(3):268-75.

105. Schmidt KL, Ernst E, Byskov AG, Nyboe Andersen A, Yding Andersen C. Survival of primordial follicles following prolonged transportation of ovarian tissue prior to cryopreservation. Hum Reprod. 2003;18(12):2654-9.

106. Donnez J, Dolmans M, Demylle D, Jadoul P, Pirard C, Squifflet J, et al. Livebirth after orthotopic transplantation of cryopreserved ovarian tissue. The Lancet. 2004;364(9443):1405-10.
